# Orphan nuclear receptor *NR2E3* is a new molecular vulnerability in solid tumors by activating p53

**DOI:** 10.1038/s41419-025-07337-1

**Published:** 2025-01-14

**Authors:** Yidan Wang, Todd G. Kroll, Linhui Hao, Zhi Wen

**Affiliations:** 1 McArdle Laboratory for Cancer Research, University of Wisconsin-Madison, Madison, WI USA; 2 Department of Pathology, Marshfield Medical Center, Marshfield Clinic Health System, Marshfield, WI USA; 3 Department of Pathology and Laboratory Medicine, Endeavor Northshore Health System, Evanston, IL USA; 4 Institute of Molecular Virology, University of Wisconsin-Madison, Madison, WI USA; 5 Center for Precision Medicine Research, Marshfield Clinic Research Institute, Marshfield Clinic Health System, Marshfield, WI USA

**Keywords:** Drug development, Tumour-suppressor proteins

## Abstract

The orphan nuclear receptor NR2E3 has emerged as a potential tumor suppressor, yet its precise mechanisms in tumorigenesis require further investigation. Here, we demonstrate that the full-length protein isoform of NR2E3 instead of its short isoform activates wild-type p53 and is capable of rescuing certain p53 mutations in various cancer cell lines. Importantly, we observe a higher frequency of NR2E3 mutations in three solid tumors compared to the reference population, highlighting its potential significance in tumorigenesis. Specifically, we identify a cancer-associated NR2E3^R97H^ mutation, which not only fails to activate p53 but also impedes NR2E3^WT^-mediated p53 acetylation. Moreover, we show that the small-molecule agonist of NR2E3, 11a, penetrates tumor mass of uterine cancer patients and increases p53 activation. Additionally, both NR2E3 and 11a exhibit similar multifaceted anti-cancer properties, underscoring NR2E3 as a novel molecular vulnerability in cancer cells. We further explore drug repurposing screens of FDA-approved anti-cancer drugs to develop NR2E3-targeted combinatorial treatments, such as the 11a-Romidepsin combination in HeLa cells. The underlying molecular mechanisms of these drug synergies include the activation of p53 pathway and inhibition of oncogenic pathway like MYC. Overall, our findings suggest that NR2E3 holds promise as a therapeutic target for cancer treatment, offering new avenues for effective anti-cancer strategies.

## Introduction

Orphan Nuclear Receptor subfamily 2 group E member 3 (NR2E3) was initially cloned from human retinoblastoma Y79 cells [1] and a mouse λZAP eye cDNA library [2] in 1999. The human NR2E3 allele is located between q22.33-->q23 on chromosome 15, while in mice, it resides on chromosome 9 [3]. Human NR2E3 has two protein isoforms, whereas mice have one. The full-length human NR2E3 comprises 410 amino acids, containing a DNA-binding domain (DBD Cys47-Val130) and a Ligand-binding domain (LBD His221-Asn410) [4]. The crystal structure of the LBD reveals a dimeric auto-repressed conformation [5]. Conversely, the short isoform lacks the C-terminal 43 amino acids. NR2E3 acts as both a transcription factor, binding to a consensus DNA duplex site (AAAGTCAAAAGTCA) [1, 6], and a transcriptional co-factor [7]. While NR2E3’s natural ligands remain unknown, small compounds like 11a, featuring a 2-phenylbenzimidazole core, act as potent agonists of NR2E3 transactivity [8].

NR2E3 mutations are implicated in various inherited retinal degenerations, including Enhanced S-cone syndrome [9]. Deletion of a 380-nt fragment in the mouse *Nr2e3* coding region disrupts the DNA-binding domain (DBD), leading to similar retinal degenerations observed in the rd7 mouse model [10]. These conditions entail the loss of rod photoreceptors and the acquisition of cone photoreceptors [11]. Interestingly, dysplastic changes occur in human retinal degenerations with mutated NR2E3, indicating a previously unrecognized proliferative response [12]. Additionally, retinal dysplasia and dysregulation of *Survivin*, a gene repressed by p53, are observed in rd7 mice [10, 13], suggesting a role for NR2E3 in regulating both cell proliferation and survival. These findings underpin the significance of NR2E3 mutations identified in cancer samples.

p53 serves as a critical tumor suppressor by inducing cell apoptosis and other essential functions [14–16]. Dysfunctional p53, resulting from mutations and altered post-translational modifications like acetylation, is prevalent in various cancers [17, 18]. For instance, the high-risk Human Papilloma Virus oncoprotein E6 facilitates p53 ubiquitination and subsequent proteasomal degradation, a pivotal step in cervical cancer tumorigenesis [19]. In our previous study, NR2E3 emerged as a host factor that activates p53 in E6^+^ cervical cancer HeLa cells, identified through a high-throughput screen of 16,000 human and mouse cDNAs [20]. NR2E3 promotes p300/PCAF-mediated acetylation of p53, enhancing cancer cell apoptosis [20]. Sensitivity to NR2E3’s agonist 11a was observed in cell lines harboring wild-type p53 within the NCI-60 cancer cell panel [21]. Additionally, activation of p53 by NR2E3 via the long non-coding RNA DINO was reported [22]. Together with positive associations between NR2E3 and recurrence-free survival in ER^+^ breast cancer and overall survival in liver cancer [23, 24], these findings suggest NR2E3’s potential role as a tumor suppressor. In the current study, we focused on the interactions between wild-type NR2E3, NR2E3 mutations, NR2E3’s agonist 11a, and p53 to enhance our understanding of NR2E3 as a novel molecular target in cancer.

## Methods and materials

Please see the supplementary files.

## Results

### NR2E3 activates p53 to inhibit solid tumor cells

**Fig. 1:**
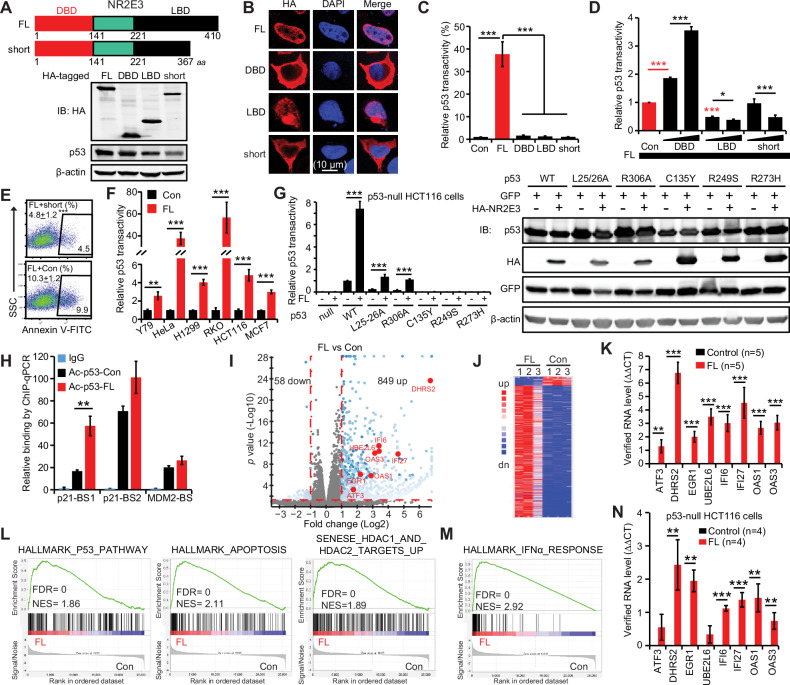
NR2E3’s full-length isoform instead of its short isoform activates p53 and suppresses cancer cells. **A** The short failed to increase p53 protein level in HeLa cells two days after transfection with 1.2 µg of the indicated constructs in 6-cm dishes. *Up*: illustration of human NR2E3 proteins. DBD: DNA binding domain; LBD ligand binding domain, FL full-length isoform; and short: short-length isoform. *Low*: immunoblotting analysis of HA-tagged NR2E3 constructs and p53. **B** Identification of a nuclear localization signal at the C-terminus of NR2E3. Immunofluorescence was conducted with confocal microscopy and anti-HA antibody in HeLa cells two days after transfection with 0.02 µg of the indicated constructs in 8-chamber slides. **C** The short failed to activate the p53RE-FLuc reporter in HeLa cells two days after transfection with 0.01 µg of the indicated constructs in 96-well plates. SV40-RLuc was used as the transfection reference. The relative FLuc activity in the empty vector control was normalized to 1. *p* value was calculated by one-way Anova test and Bonferroni post-hoc test (each domain vs Con vs FL). *p < 0.0167; **p < 0.0033; ***p < 0.0003. **D** The short inhibited the FL’s activation of the p53RE-FLuc reporter in HeLa cells two days after transfection with both 0.01 µg FL and 0.01 µg or 0.03 µg of the indicated constructs in 96-well plates. SV40-RLuc was used as the transfection reference. The relative FLuc activity in the FL+empty vector control was normalized to 1. red *p values of FL+empty vector control vs low dose. *p* value was calculated by one-way Anova test and Bonferroni post-hoc test (FL vs low dose vs high dose). *p < 0.0167; **p < 0.0033; ***p < 0.0003. **E** The short inhibited the FL-induced HeLa cell apoptosis two days after transfection with both 1.2 µg FL and 2.8 µg of short or empty vector in 6-cm dishes in Annexin V-binding assay. *p* value was calculated by Student’s t-test with two tails. ***p < 0.001. **F** The FL activated the p53RE-FLuc reporter in six cancer cell lines two days after transfection with 0.01 µg FL in 96-well plates. These cells endogenously express wild-type p53, except H1299 cells with enforced p53 expression. The relative FLuc activity in mock control was normalized to 1. *p* value was calculated by Student’s t-test with two tails. **p < 0.01; ***p < 0.001. **G** The FL selectively rescued the wild-type transactivities of p53 mutations which partially reserved this transactivity in p53^-/-^ HCT116 cells. 0.01 µg of the indicated p53 constructs was co-transfected with the p53RE-FLuc reporter, SV40-RLuc and 0.01 µg FL/empty vector in 96-well plates. The relative FLuc activity in the p53^WT^+empty vector control was normalized to 1. *p* value was calculated by Student’s t-test with two tails. ***p < 0.001. *Left:* Immunoblotting assay showed the protein levels of p53 mutations. *GFP*: transfection reference. **H** The FL enhanced the binding of acetylated p53 to p21 promoter in HeLa cells two days after transfection with 1.2 µg p53 plasmid and 2.4 µg FL in 10-cm dishes. Anti-acetylated p53 antibody was used in chromatin immunoprecipitation (ChIP) followed by qPCR analysis. *BS:* p53-binding site. **I**−**M** RNA-sequencing analysis of HeLa cells two days after transfection with 2 µg FL or empty vector. **I** Volcano plot of all transcripts. *Red:* growth inhibitory genes. **J** Heat map of the most differentially expressed genes. **K** Up-regulations of growth inhibitory genes were verified by qRT-PCR. *p* value was calculated by Student’s t-test with two tails. **p < 0.01; ***p < 0.001. **L** Gene sets of p53, apo*p*tosis and HDAC were enriched in FL group. **M** Gene set of INFα was enriched in FL grou*p*. **N** RNA levels of growth inhibitory genes were measured by qRT-PCR in p53^-/-^ HCT116 cells two days after transfection with 4 µg FL in 6-cm dishes. *p* value was calculated by Student’s t-test with two tails. ^**^p < 0.01; ***p < 0.001. Mean ± SD shown in all the histograms.

In addition to retinal photoreceptor cells, NR2E3 is expressed in many tissues such as the urogenital and respiratory systems in humans and mice, suggesting potential functions beyond the retina (supplementary Fig. S1). With the recently available anti-NR2E3 antibodies, the protein of NR2E3 has been detected in quite a few tissues and cell lines of both human and mouse [23, 24]. To further elucidate NR2E3’s roles in cancer, we expressed HA-tagged NR2E3 protein derivatives, including the full-length isoform (FL), DNA-binding domain (DBD), ligand-binding domain (LBD), and short isoform (short), in HeLa cells (Fig. 1A). Interestingly, while the FL localized to the nucleus as a transcription factor, the short isoform remained outside the nucleus, indicating a nuclear localization signal in the FL’s C-terminal 43 amino acids. Consistent with this, the DBD was cytosolic, and the LBD was nuclear (Fig. 1B). Indeed, both the short and DBD failed to stimulate p53 transactivity, possibly due to their cytosolic localization (Fig. 1C). The LBD did not stimulate p53 transactivity either because of its inability to increase p53 (Fig. 1A). Surprisingly, when co-expressed with the FL, the DBD enhanced the FL-mediated p53 transactivity, possibly through forming a dimer with the FL which is a common function of DBD in Nuclear Receptor family [25] while the LBD inhibited it, potentially by competing with the FL for its unknown ligand (Fig. 1D). Surprisingly, the short exerted dominant-negative effects in p53 reporter and cell apoptosis assays (Fig. 1D, E). Since the short arises naturally from alternative RNA splicing, our findings suggest that investigating the imbalance between both isoforms may provide insights into NR2E3’s roles in cancer.

Besides in HPV^+^ HeLa cells [20], the stimulation of p53 transactivity by full-length NR2E3 (FL) was also observed in human retinoblastoma-Y79, lung cancer-H1299, colon cancer-RKO and HCT116, and breast cancer-MCF7 cells expressing wild-type p53, suggesting a conserved mechanism for NR2E3-mediated activation of p53 across at least five types of solid tumors (Fig. 1F). Additionally, NR2E3’s closely related subfamily members NR2E1, NR2F1, and NR2F2 also stimulated p53 transactivity in HeLa and HCT116 cells, indicating a shared role of these orphan nuclear receptors in activating p53 (supplementary Fig. S2). Given the prevalence of mutated p53 in cancer, we investigated the effects of five p53 mutations—L25-26A [26], C135Y, R249S, R273H, and R306A [27] —on p53 transactivity when co-expressed with the FL. While p53^L25-26A^ and p53^R306A^ partially preserved p53^WT^ transactivity, p53^C135Y^, p53^R249S^, and p53^R273H^ completely abolished it in p53^-/-^ HCT116 cells (Fig. 1G) and p53-null H1299 cells (supplementary Fig. S3A). Interestingly, the FL enhanced the wild-type transactivities of p53^L25-26A^ and p53^R306A^ but not of p53^C135Y^, p53^R249S^, and p53^R273H^, suggesting that NR2E3 can augment the residual wild-type transactivities of p53^MUT^ (Fig. 1G and supplementary Fig. S3A). The activation of transactivities of these p53^MUT^ by the FL correlated to their increased protein levels which were shown when normalized to GFP protein serving as the transfection control (Fig. 1G and supplementary Fig. S3B).

We demonstrated that NR2E3 enhances p53 acetylation, selectively promoting the up-regulation of certain p53 target genes like p21, while leaving others such as MDM2 unaffected [17, 20]. In HeLa cells, the FL facilitated acetylated p53 binding to its DNA consensus on p21 rather than MDM2 (Fig. 1H), suggesting a role for NR2E3 in p53-mediated transcriptional regulation. RNA-seq analysis in HeLa cells expressing the FL revealed up-regulation of 849 genes and down-regulation of 58 genes (fold change > 2; *p* < 0.05) (Fig. 1I, J). qRT-PCR validation confirmed the up-regulation of genes involved in apoptosis and cell growth inhibition, including UBE2L6, IFI6, IFI27, OAS1, and OAS3 (Fig. 1K), consistent with the enrichment of p53, apoptosis, and IFN-α pathways identified by gene set enrichment analysis (GSEA) (Fig. 1L, M). For example, UBE2L6, encoding the ubiquitin-conjugating enzyme UBCH8, redirects E6AP to ubiquitinate HPV E6-independent substrates [28]. It also is responsive to p53 under DNA damage conditions [29]. IFI6, IFI27, OAS1, and OAS3 are part of the interferon-alpha response (IFN-α) pathway, known for its anti-tumor and anti-viral immune responses [30]. NR2E3 also up-regulated these genes in *p53*^*-/-*^ HCT116 and p53-null H1299 cells, but the degrees of the up-regulations were significantly lower than those in HeLa cells, presenting the involvement of p53 (Fig. 1N and supplementary Fig. S4). Additionally, re-analysis of RNA microarray data from retinal cells in wild-type and *Nr2e3*-null mice (*rd7* and KO) [31] supported the tumor-suppressive role of NR2E3 via p53 (supplementary Fig. S5). Overall, these findings highlight NR2E3’s role in regulating p53, the IFN-α pathway, and other pathways to suppress tumors.

### NR2E3 mutations are implicated in solid tumors

**Fig. 2:**
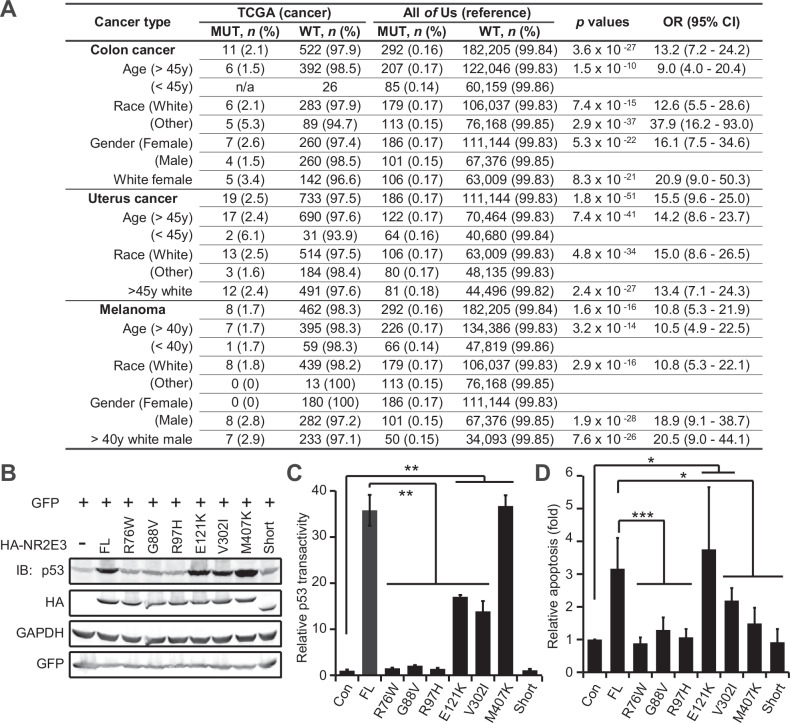
NR2E3 mutations are closely associated with cancer. **A** NR2E3 mutations were enriched in colon cancer, uterus cancer and melanoma. Nonsynonymous SNVs of NR2E3 were counted in TCGA cancer database and “All *of* Us” reference database. Age, sex and race were considered. The age group cutoffs were set [53–55]. Chi-square test (two-tail, 1 df) and Woolf logit test (n > 99k) or Baptista-Pike (n < 99k) test were used to calculate the *p* values and OR values with 95% confidence interval only in the comparisons with minimal case number of 5. **B** NR2E3 SNVs differentially regulated p53 protein levels in HeLa cells two days after transfection with 1.2 µg of the indicated constructs in 6-cm dishes in immunoblotting assay. *GFP*: transfection reference. **C** NR2E3 SNVs differentially stimulated the p53RE-FLuc reporter in HeLa cells two days after transfection with 0.01 µg of the indicated constructs in 96-well plates. SV40-RLuc was used as the transfection reference. The relative FLuc activity in the empty vector control was normalized to 1. *p* value was calculated by one-way Anova test and Bonferroni post-hoc test (each mutation vs Con vs FL). *p < 0.0167; **p < 0.0033; ***p < 0.0003. **D** NR2E3 SNVs differentially regulated HeLa cell apoptosis two days after transfection with 1.2 µg of the indicated constructs in 6-cm dishes in Annexin V-binding assay. The apoptosis in the mock control was normalized to 1. *p* value was calculated by one-way Anova test and Bonferroni post-hoc test (each mutation vs Con vs FL). *p < 0.0167; **p < 0.0033; ***p < 0.0003. Mean ± SD shown in all the histograms.

We compiled data on over 500 disease-associated single nucleotide variants (SNVs) or mutations of NR2E3, primarily annotated in ClinVar (supplementary Fig. S6A). Notably, these variants were exclusively germline SNVs. In our analysis of cancer cases from The Cancer Genome Atlas (TCGA), we observed NR2E3 mutations altering the protein sequence. Specifically, we found 11/522 (NR2E3 mutation cases *vs* WT cases) in colon cancer, 19/733 in uterine cancer, and 8/462 in melanoma (Fig. 2A). Significant associations between NR2E3 mutations and these cancer types were evident compared to the reference population in the “All of Us” database which encompasses diverse demographic groups (*p* < 0.05, high OR with 95% CI, adjusting for race, sex, and age) (Fig. 2A). Our findings underpin NR2E3 mutations as potential risk factors for these solid tumors.

Most of the NR2E3 SNVs reported in clinical specimens lacked experimental characterization, leading ClinVar to computationally categorize them as “benign,” “pathogenic,” or “uncertain” (supplementary Fig. S6B). Stratifying over 500 NR2E3 SNVs using experimental tools was crucial, which we achieved using the p53 reporter assay. In a pilot test, we selected six disease-associated SNVs previously examined in human and monkey kidney cells [32]. Notably, two patients with uterine corpus endometrial carcinoma in the TCGA cohort harbored the R97H mutation (supplementary Fig. S6C). Comparing to the FL, pathogenic R76W, G88V, and R97H did not increase p53 protein levels, while benign E121K and V302I and uncertain M407K did (Fig. 2B). Consistent with the p53 protein levels, we observed activation of the p53 reporter by E121K, V302I, and M407K, but not by R76W, G88V, and R97H, in HeLa, H1299, and HCT116 cells (Fig. 2C and supplementary Fig. S7). Consequently, R76W, G88V, and R97H did not enhance cell apoptosis, while E121K and V302I induced cell apoptosis (Fig. 2D and supplementary Fig. S8). In summary, we provided solid evidence supporting ClinVar’s predictions and developed an easy assay using the p53 reporter to stratify NR2E3 SNVs for their implications in cancer.

### Pathogenic R76W and R97H mutations fail to activate p53 as loss-of-function mutations

**Fig. 3:**
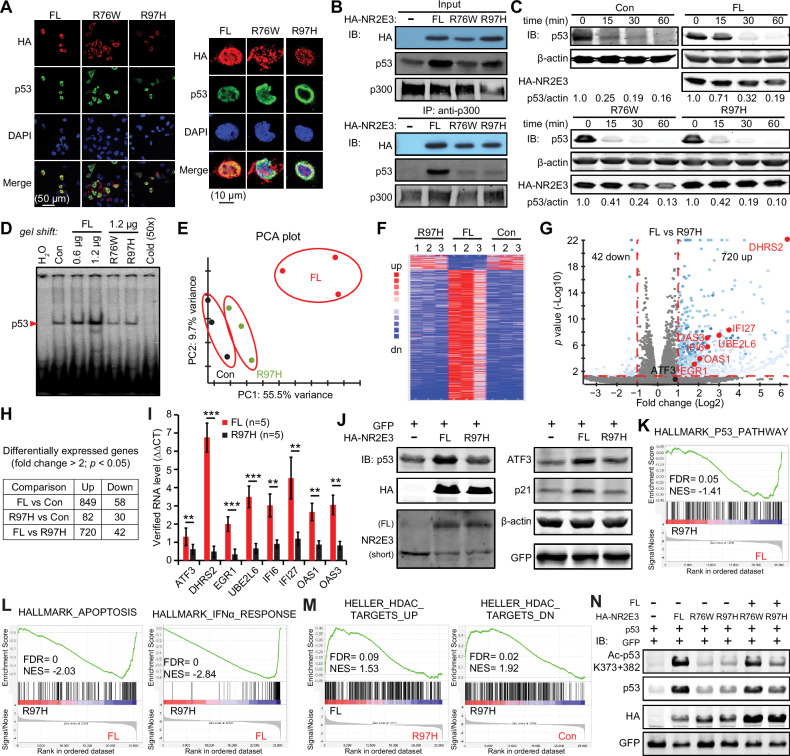
NR2E3 R76W and R97H are loss of function mutations. **A** Immunofluorescence analysis of co-localization of p53 and FL/R76W/R97H in HeLa cells two days after transfection with 0.02 µg of the indicated constructs in 8-chamber slides. Images were collected with confocal microscopy. *Left:* zoom-in images for the details. **B** R76W and R97H failed to enhance p300’s binding to p53 in co-immunoprecipitation assay with an anti-p300 antibody followed by immunoblotting analysis. 0.5 µg p53, 1.5 µg p300 and 1.5 µg FL/R76W/R97H were co-transfected into HeLa cells in 6-cm dishes for two days. **C** R76W and R97H failed to extend half-life time of p53 protein in HeLa cells two days after transfection with 1.2 µg of the indicated constructs in 6-cm dishes. The protein synthesis was ceased by 2 µg/mL of Cycloheximide and the cell lysates were collected at the indicated treatment time. **D** R76W and R97H failed to enhance HeLa cell nuclear extract’s binding to p53-DNA consensus in gel shift analysis. Cold (unlabeled) probe was loaded 50-fold more than the ^32^P-labeled probe. The indicated amount of plasmids were transfected into HeLa cells in 6-cm dishes for two days. **E**−**L** RNA-sequencing analysis of HeLa cells two days after transfection with 2.0 µg of the indicated constructs in 6-cm dishes. **E** Principal Component Analysis (PCA) plot of 9 samples. **F** Heat map of the most differentially expressed genes in FL, R97H and control groups. **G** Volcano plot of the transcripts of FL and R97H groups. *Red:* growth inhibitory genes. **H** The table summarized the number of differentially expressed genes in each comparison. **I** Expressions of growth inhibitory genes in (**G**) were verified using qRT-PCR. Mean ± SD shown. *p* value was calculated by Student’s t-test with two tails. **p < 0.01; ***p < 0.001. **J** Immunoblotting assay showed that R97H lost the ability to increase protein levels of p53, ATF3 and p21 in HeLa cells two days after transfection with 2.0 µg of the indicated constructs in 6-cm dishes. **K**−**M** GSEA studies of RNA-sequencing data. p53 (**K**), apoptosis and IFN-α (**L**) pathways were enriched in FL group. **M** Acetylation-related gene sets were enriched in FL group while Deacetylation-related pathway was enriched in R97H group. **N** R97H abolished the FL from acetylating p53 in the immunoblotting assay. HeLa cells were transfected with 0.6 µg p53, 0.2 µg GFP and 2.0 µg FL/R76W/R97H in 6-cm dishes for two days. *GFP*: transfection reference.

Unlike the FL, the R76W and R97H variants localize in both the cytoplasm and nucleus in HEK293 cells [32]. We observed similar subcellular localizations of R76W and R97H in HeLa cells, with much less co-localization between p53 and R76W/R97H compared to the FL (Fig. 3A). We previously reported that NR2E3^WT^ forms a complex with p300 and p53 as a transcriptional co-activator to enhance p53 acetylation by p300 [20]. Co-immunoprecipitation with the anti-p300 antibody showed similar associations of the FL, R76W, and R97H with p300, indicating that these mutations did not disrupt their interactions with p300 (Fig. 3B). However, R76W and R97H failed to increase the association between p300 and p53, as observed with the FL (Fig. 3B). Furthermore, the extended half-life time of p53 protein observed with the FL [20] was not seen in the R76W and R97H groups (Fig. 3C). Gel-shift assays using a ^32^P-labeled probe containing the classic p53-binding consensus sequence revealed that while the FL enhanced p53-probe binding in a dose-dependent manner, R76W and R97H did not induce this enhancement even when enforcedly expressed at the high doses (Fig. 3D). Together, these findings indicate that R76W and R97H are loss-of-function mutations regarding their ability to activate p53.

We selected the cancer-associated R97H for transcriptomic studies to further determine its functions. Overall, R97H behaved very similarly to the mock control, indicating it is a loss-of-function mutation (Fig. 3E−G). For instance, eight genes in the p53 and IFN-α pathways, which were up-regulated by the FL, were only mildly affected by R97H (Fig. 3H, I). We verified the similar changes at the protein levels of ATF3 and p21 (Fig. 3J). Comparing R97H to the FL, it failed to enhance p53 pathways (Fig. 3K), indicating that R97H loses the wild-type functions needed to activate p53. Similarly, R97H did not activate the IFN-α pathway or induce apoptosis like its FL counterpart (Fig. 3L).

We further analyzed R97H’s capability to enhance p53 acetylation. While the FL enriched the gene set up-regulated by the HDAC inhibitor Trichostatin A, supporting its role in enhancing acetylation, R97H increased the genes down-regulated by Trichostatin A, suggesting a potential HDAC function (Fig. 3M). Consistent with GSEA results, R76W and R97H did not enhance p53 acetylation, displaying a loss of function (Fig. 3N). Additionally, R97H inhibited the FL-mediated p53 acetylation, indicating a dominant-negative function. These findings provide mechanistic insights into the association between NR2E3 mutations and cancer (Fig. 2A).

### NR2E3 agonist 11a promotes NR2E3 to activate p53

Additionally, the statistical tool GEPIA [33] correlated *NR2E3* RNA levels with the overall and disease-free survivals of cancer patients in the TCGA database. Regardless of the FL or short, high *NR2E3* RNA levels when normalizing to β-actin were linked to superior disease-free and overall survivals across 33 cancer types when combined (supplementary Fig. S9A, B). Despite that the short is dominant-negative to the FL, the short’s associating with superior prognosis may be the consequence of enhanced transcription of *NR2E3* gene, instead of modified alternative splicing. To investigate the imbalance between the FL and the short, we normalized the FL RNA level to the short RNA level for survival survey, showing that the high FL vs short ratio was correlated to the superior disease-free and overall survivals (supplementary Fig. S9C, D). These findings, coupled with previously published associations between NR2E3 expression levels and prognosis in breast cancer and liver cancer [22–24], reaffirm that elevated NR2E3 expression predicts better cancer prognosis. This underscores the potential therapeutic value of activating NR2E3 in cancer treatment.

**Fig. 4:**
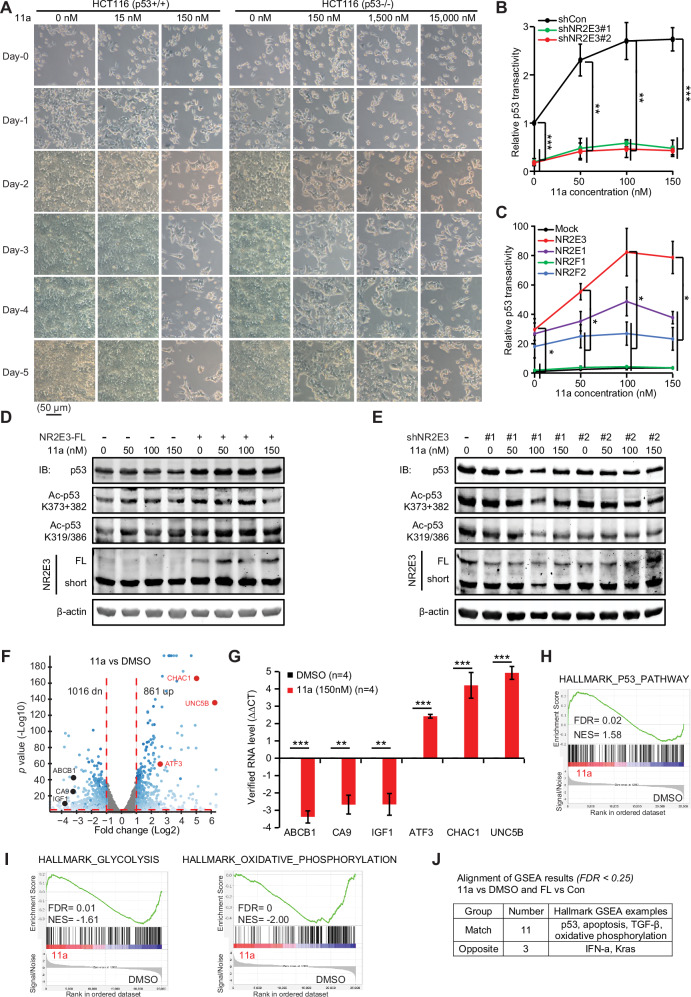
NR2E3’s agonist 11a specifically enhances NR2E3 to activate p53 in HeLa cells. **A** 11a has a stronger inhibition of *p53*^*+/+*^ HCT116 cells than *p53*^*-/-*^ HCT116 cells. The same number of *p53*^*-/-*^ and *p53*^*+/+*^ isogenic HCT116 cells were seeded in 6-well plates and the indicated concentrations of 11a were achieved in each well. Photos of these cells were taken daily from day-0 to day-5. **B** shNR2E3 disrupted the 11a’s stimulation of the p53 reporter. 0.06 µg of the indicated shRNAs were co-transfected with the p53RE-SEAP reporter and SV40-RLuc into HeLa cells in 96-well plates. One day after transfection, 11a was added to achieve the indicated concentrations in each well. One day after 11a treatment, the luminescence was measured. The relative SEAP activity in the DMSO+scramble shRNA control was normalized to 1. *p* value was calculated by one-way Anova test and Bonferroni post-hoc test (shCon vs two shNR2E3). *p < 0.025; **p < 0.005; ***p < 0.0005. **C** 11a activated the p53 reporter much more in NR2E3 group than other groups. 0.01 µg of NR2E3 or its subfamily members were co-transfected with the p53RE-SEAP reporter and SV40-RLuc into HeLa cells in 96-well plates. One day after transfection, 11a was added to achieve the indicated concentrations in each well. One day after 11a treatment, the luminescence was measured. The relative SEAP activity in the DMSO control in mock control was normalized to 1. *p* value was calculated by one-way Anova test and Bonferroni post-hoc test (NR2E3 vs others). *p < 0.0125; **p < 0.0025; ***p < 0.00025. **D** HeLa cells were transfected with 1.2 µg FL or mock control in 6-cm dishes. 11a was added with fresh medium one day after transfection. Immunoblotting assays showed that one day treatment with 11a increased the acetylation of endogenous p53 at K319/386 in the control group (*left*). When HeLa cells were overexpressing the FL, immunoblotting assays showed the enhanced p53 acetylation at K319/386 and total protein level (*right*). One day treatment with 11a further increased the acetylation of p53 at K319/386. **E** HeLa cells were transfected with 3.8 µg shRNAs against NR2E3 or mock control in 6-cm dishes. 11a was added with fresh medium one day after transfection. Immunoblotting assays showed that knocking down NR2E3 prevented 11a from increasing the p53 acetylation at K319/386. **F**−**J** RNA sequencing was conducted in HeLa cells treated with 150 nM 11a or DMSO for one day. **F** Volcano plot of all transcripts of 11a and DMSO control. *Red:* growth inhibitory genes; *Black:* growth-promoting genes. **G** Changes of the selected genes were verified using qRT-PCR. *p* value was calculated by Student’s t-test with two tails. **p < 0.01; ***p < 0.001. **H** p53 pathway was enriched by 11a. **I** Metabolic glycolysis and oxidative phosphorylation pathways were enriched in DMSO controls. **J** Comparison of GSEA results between 11a vs DMSO and FL vs Control. Mean ± SD shown in all the histograms.

Small compound 11a, featuring a 2-phenylbenzimidazole core, acts as an agonist of transcription factor NR2E3 which recruits its co-repressors to repress the transcriptions of cone-specific genes in rod photoreceptor cells in eye [8, 34]. While we previously observed that cell lines harboring wild-type p53 are more sensitive to 11a compared to those with p53 mutations in the NCI-60 cancer cell panel [21], the hypothesis that 11a’s anti-tumor effects rely on the transcription factor activity of NR2E3 and the following molecular mechanism study conducted with a DR2-reporter and cell lines bearing p53 mutations were not well justified (see *supplementary discussion* for details). Here, we found that *p53*^*+/+*^ HCT116 cells exhibited at least 100-fold greater sensitivity to 11a compared to their *p53*^*-/-*^ counterparts, indicating a dependency of 11a on wild-type p53 (Fig. 4A). Together with NR2E3’s stimulating p53 acetylation as a transcriptional co-activator [20], we selected the p53 reporter to study how 11a stimulates transcription factor p53 via p53’s transcriptional co-activator NR2E3, instead of the DR2-reporter which is neither a validated NR2E3 reporter nor a p53 reporter. 11a activated the p53 reporter in HeLa cells expressing wild-type p53, an effect abolished upon NR2E3 knockdown (Fig. 4B), suggesting that 11a can enhance the activity of NR2E3 as the transcriptional co-activator of p53 transactivity [20]. Moreover, 11a induced greater activation of the p53 reporter in cells overexpressing NR2E3 compared to those overexpressing NR2E1, NR2F1, or NR2F2, underscoring its heightened bioactivity for NR2E3-mediated p53 transactivation (Fig. 4C).

11a increased the acetylation at K319/386 of endogenous p53 while its regulation of the acetylation at K373 + 382 was not sound in HeLa cells (Fig. 4D, *left*). We enforced the expressions of the FL in HeLa cells which were then treated with 11a. The FL increased the protein level and acetylation at K319/386 of endogenous p53 (Fig. 4D, *right*). 11a further enhanced the acetylation at K319/386 of endogenous p53, (Fig. 4D, *right*). When endogenous NR2E3 was knocked down by two shRNAs in HeLa cells, the acetylation at K319/386 of endogenous p53 was also decreased (Fig. 4E). The 11a-induced increases of acetylation at K319/386 of endogenous p53 were markedly repressed (Fig. 4E). In contrast, the overexpression and knockdown of NR2E3 slightly regulated the acetylation at K373 + 382 alone or combined with 11a treatment. In *p53*^*+/+*^ HCT116 cells, we detected both isoforms of endogenous NR2E3 with the recently available anti-NR2E3 antibody (supplementary Fig. S10), which was not detected with a home-made NR2E3 antibody in our previous study [21]. Despite that the protein level of endogenous p53 was not significantly modified by 11a, the acetylations at K319/373/382/386 of p53 were enhanced by 11a, leading to the increases of p53 targets p21, ATF3 and Puma in *p53*^*+/+*^ HCT116 cells (supplementary Fig. S10). Collectively, our data showed that 11a can enhance the NR2E3-mediated p53 acetylation.

We conducted RNA-seq assays to investigate the anti-cancer effects of 11a in HeLa cells (Fig. 4F). The expression patterns of six cancer-associated genes were validated using qRT-PCR (Fig. 4G), including the down-regulation of ABCB1, a crucial ATP-dependent drug efflux pump involved in drug resistance across various cancer types [35], as well as the up-regulations of several pre-apoptotic genes in the p53 pathway (*e.g*., CHAC1, UNC5B, and ATF3) (Fig. 4G, H) as NR2E3 did (Fig. 1K). 11a also suppressed both the oxidative phosphorylation and glycolysis pathways (Fig. 4I). Alignment of GSEA results in human Hallmark collection between 11a vs DMSO and FL vs Control revealed that 11a shares similar roles with NR2E3, supporting its role as a NR2E3 agonist (Fig. 4J). Re-analysis of RNA microarray data [31] demonstrated the anti-cancer effects of 11a in suppressing the NF-κB pathway in C57BL/6 mouse retinal tissue compared to the DMSO control (supplementary Fig. S11). Overall, our findings suggest that 11a exerts multifaceted anti-cancer effects, mainly including the activation of p53 and NR2E3.

### Developing NR2E3-targeted combinatorial therapies in solid tumors

**Fig. 5:**
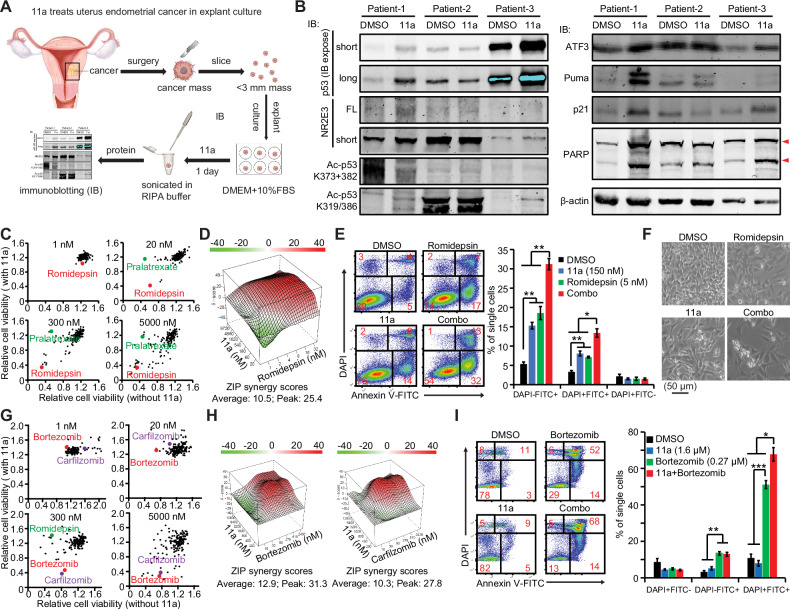
Identify NR2E3-targeted combinatorial treatments in solid tumors. **A** Illustration of treating human uterine endometrial cancer with 11a in explant culture. **B** Immunoblotting assays showed that one day treatment with 4 µM 11a increased p53 protein level, stimulated p53 acetylation at K319/386, activated p53-targeted genes, and induced cell apoptosis signaling in 2 out of 3 patient samples. **C** Repurposing screens of FDA-approved anti-cancer drug library (NCI AOD X version) presented Romidepsin as a lead to promote 11a’s cell-killing effect in cervical cancer HeLa cells. Four concentrations (1 nM, 20 nM, 300 nM and 5 µM) of each drug in the library were achieved when mixed with 50 µL of cell suspensions and one concentration of 11a (1.5 µM) were tested in 384-well plates. The cell viability was measured by CellTiter-Glo in two days and the DMSO control in each plate was normalized to 1. The normalized cell viabilities in the presence of 11a (y-axis) were plotted against those in the absence of 11a (x-axis) for each concentration. **D** ZIP drug synergy score showed the synergy between 11a and Romidepsin. 8 × 9 doses of 11a and Romidepsin were mixed with HeLa cells in 96-well plates for two days. The cell viability of DMSO control was normalized to 100%. The data were evaluated for synergy by ZIP score [39]. **E** The 11a-Romidepsin combo induced more HeLa cell apoptosis than each individual drug in one day in Annexin V-binding assay. *p* value was calculated by one-way Anova test and Bonferroni post-hoc test (DMSO vs 11a vs Romidepsin vs Combo). *p < 0.0083; **p < 0.00167; ***p < 0.00017. **F** The combo had much stronger inhibition of HeLa cell growth than each individual drug in two days. **G** Repurposing screens of AOD X library as done in (**A**) identified Bortezomib and Carfilzomib promoting 11a’s cell-killing effects in retinoblastoma Y79 cells. **H** ZIP drug synergy assay as (**D**) showed the synergies between 11a and Carfilzomib or Bortezomib in Y79 cells. **I** The 11a-Bortezomib combo induced more Y79 cell apoptosis than each individual drugs in one day in Annexin V-binding assay. *p* value was calculated by one-way Anova test and Bonferroni post-hoc test (DMSO vs 11a vs Romidepsin vs Combo). *p < 0.0083; **p < 0.00167; ***p < 0.00017. Mean ± SD shown in all the histograms.

First, we examined the cell-killing effect of 11a as a single anti-cancer agent in the explant culture of human endometrial cancer specimens which reserved the 3-D histological architecture of tumor (Fig. 5A). We observed the increases of p53 protein levels and acetylations at K319/386 in 2 out of 3 cases, which up-regulated ATF3, Puma, p21 and PARP cleavage in the p53 and cell apoptosis pathways (Fig. 5B). Of note, the patient-2 expressed the short isoform of NR2E3 at a much higher level than the patient-1 and -3. As the short behaved dominantly-negative to the FL (Fig. 1A−E), the patient-2 showed no response to 11a treatment. Collectively, our result showed that 11a is able to penetrate the solid tumor to agonize NR2E3, which is of a great value as an anti-cancer drug candidate.

Generally speaking, combinatorial therapy is superior to single-agent therapy by simultaneously targeting multiple oncogenic pathways and overcoming the resistance to one drug in most cancer treatments. We observed that HeLa cells activated alternative survival pathways such as the TGF-β pathway [36] to counteract the effects of 11a treatment and high NR2E3 expression, which necessitates combinatorial therapies (Fig. 4J). Combining with FDA-approved drug may also help to decode the mechanisms underlying drug resistance since all the drugs have been well characterized at molecular level. Therefore, we conducted drug repurposing screens in an FDA-approved anti-cancer drug library (NCI AOD X version) [37] in human cervical cancer HeLa cells and human retinoblastoma Y79 cells, both of which exhibit NR2E3 expression. Romidepsin, a potent HDAC-1/2 inhibitor (HDACi), synergized with 11a to inhibit HeLa cells, while Pralatrexate, a folate analog, counteracted 11a (Fig. 5C). Additional HDACi and DNA intercalating agents were also identified as leads, five of which, including Romidepsin, have been used or tested in clinical trials for treating cervical cancer [38]. HDACi prevents HDACs from removing acetyl groups from proteins, potentially stabilizing NR2E3-enhanced p53 acetylation. Therefore, we utilized the ZIP drug synergy scoring tool [36, 37] to evaluate the 11a-Romidepsin combination, obtaining an average ZIP score of 10.5 and a peak score of 25.4 (Fig. 5D). An ‘ZIP Average Score > 10’ suggests overall synergy between drugs [39], while the ‘ZIP Peak score’ indicates the highest synergy and optimal dosage combination. Our data indicate that 11a synergizes with Romidepsin, and the optimal dosage combination falls within their low dosage ranges. Considering the toxicities of anti-cancer drugs, combinations of low doses are preferable. Indeed, a combination from the low dosage range induced significantly more cell apoptosis than single treatments within one day (Fig. 5E). While 11a or Romidepsin alone inhibited cell growth, the combination effectively eradicated most cells within two days (Fig. 5F).

The drug repurposing screen in retinoblastoma Y79 cells, from which NR2E3 was first cloned [1], revealed that two proteasome inhibitors, Bortezomib and Carfilzomib, synergized with 11a to induce cell death at higher concentrations (Fig. 5G). Subsequent ZIP drug synergy scoring assays and Annexin V-staining assays confirmed the synergy between 11a and Bortezomib/Carfilzomib in killing Y79 cells (Fig. 5H, I). Bortezomib has been shown to induce caspase-dependent apoptosis in Y79 cells at clinically achievable concentrations [40], suggesting its potential for treating retinoblastoma. Conversely, 11a exhibited antagonism with Romidepsin in inhibiting Y79 cells (Fig. 5G). Subsequent ZIP drug synergy assays validated this antagonism in Y79 cells (*data not shown*). These differential behaviors of the 11a-Romidepsin combo between Y79 and HeLa cells were not surprising because Y79 and HeLa cells are derived from two types of cancer with markedly different histological origins and tumorigenesis. Overall, our findings offer promising combinatorial treatments targeting NR2E3 in cervical cancer and retinoblastoma cells, highlighting NR2E3 as a novel molecular vulnerability in solid tumors.

### Multifaceted drug synergies between 11a and Romidepsin

**Fig. 6:**
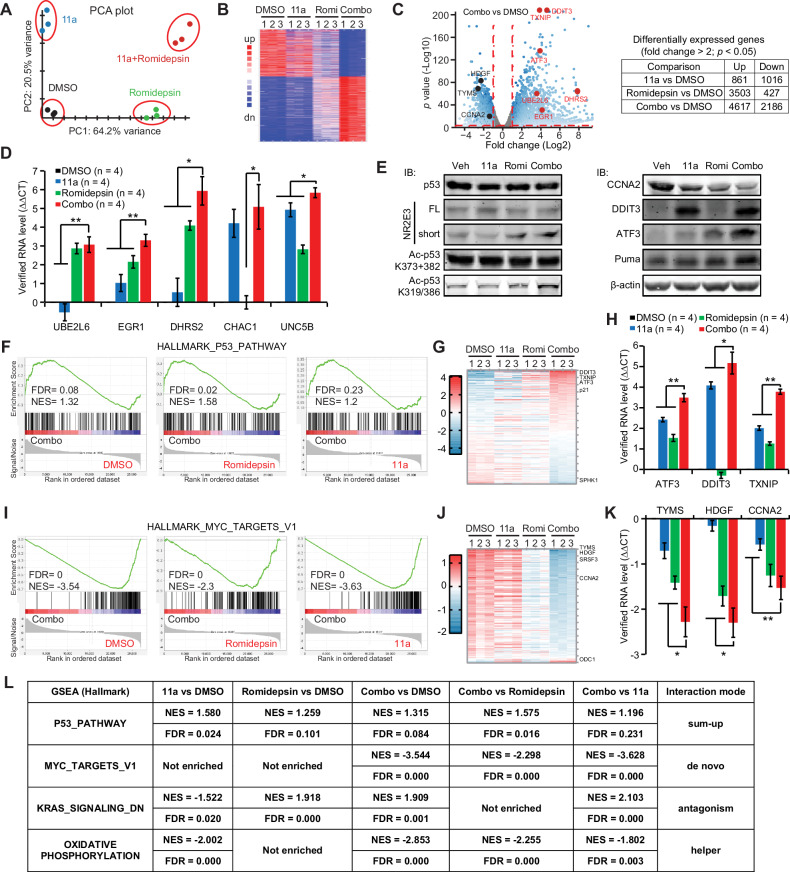
Dissecting molecular mechanisms underlying the drug synergy of 11a and Romidepsin. RNA-sequencing analysis of HeLa cells treated with DMSO, 150 nM 11a, 5 nM Romidepsin, or the combo for one day. **A** PCA plot indicated four well-separated sample clusters. **B** Heat map of the most differentially expressed genes in four groups. **C** Volcano plot of the transcripts of the combo vs DMSO. *Red*: growth inhibitory genes and *black*: growth promoting genes. The table summarized the number of differentially expressed genes in each comparison. **D** Up-regulations of five growth inhibitory genes were verified using qRT-PCR. *p* value was calculated by one-way Anova test and Bonferroni post-hoc test (DMSO vs 11a vs Romidepsin vs Combo). *p < 0.0083; **p < 0.00167; ***p < 0.00017. **E** Up-regulations of p53-targeted genes were examined in HeLa cells using Immunoblotting assay. HeLa cells were treated with DMSO, 150 nM 11a, 5 nM Romidepsin, or the combo for one day. **F** GSEA plots of p53 pathway in the indicated comparison. **G** Heat map of differentially expressed genes in the p53 pathway among 4 groups. **H** Up-regulations of p53 pathway genes were verified using qRT-PCR. *p* value was calculated by one-way Anova test and Bonferroni post-hoc test (DMSO vs 11a vs Romidepsin vs Combo). *p < 0.0083; **p < 0.00167; ***p < 0.00017. **I** GSEA plots of MYC pathway in the indicated comparison. **J** Heat map of differentially expressed genes in the MYC pathway among 4 groups. **K** Down-regulations of MYC pathway genes were verified using qRT-PCR. *p* value was calculated by one-way Anova test and Bonferroni post-hoc test (DMSO vs 11a vs Romidepsin vs Combo). *p < 0.0083; **p < 0.00167; ***p < 0.00017. **L** Four drug synergy modes were identified by multiple comparisons of GSEA. Mean ± SD shown in all the histograms.

The RNA-seq data exhibited well-separated sample clusters, supporting the synergy between 11a and Romidepsin in HeLa cells (Fig. 6A, B). The up-regulations of five tumor-suppressing genes were confirmed by qRT-PCR (Fig. 6C, D). Interestingly, Romidepsin increased the transcription of Multidrug Resistance Protein ABCB1 in HeLa cells, a phenomenon counteracted by 11a, thereby contributing to the observed drug synergy (supplementary Fig. S12A). We did not observe the increased protein level of endogenous p53 in HeLa cells treated by the 11a-Romidepsin combo for 24 h (Fig. 6E). The acetylation of p53 at K319/386 instead of K373 + 382 was increased in the 11a-Romidepsin combo group (Fig. 6E). Of note, the wild-type p53 has 20 Lysines that could be acetylated by various acetyltransferases such as p300, and so far, we have tested four sites with available antibodies in this study. We also detected the increases of p53 targeted genes such as DDIT3, ATF3 and Puma by the 11a-Romidepsin combo which decreased CCNA2 on the other hand (Fig. 6E).

Alignment of GSEA results of multiple comparisons between DMSO, 11a, Romidepsin, and the 11a-Romidepsin combination was conducted to understand the drug synergies. Pathways such as p53 were activated by 11a, Romidepsin, and the combination when compared to DMSO, respectively (Fig. 6F). Examination of all genes in the p53 pathway identified the most differentially expressed genes in the combination group, which were verified by qRT-PCR (Fig. 6G, H) and immunoblotting (Fig. 6E). Comparisons of the combination vs 11a and the combination vs Romidepsin also showed that the combination was a stronger activator of the p53 pathway than both 11a and Romidepsin. We named this type of synergy “sum-up” mode (Fig. 6L). In contrast, “antagonism” mode occurred when survival signals activated by one drug were antagonized by the other, resulting in cell death. For example, 11a down-regulated the KRAS-down pathway in 11a vs DMSO, but Romidepsin up-regulated these genes in Romidepsin vs DMSO (Fig. 6L). Ultimately, the combination up-regulated this set of genes in Combo vs DMSO, suggesting that Romidepsin antagonized and overweighed 11a to activate the KRAS-down pathway (Fig. 6L).

The 11a-Romidepsin combination repressed the MYC pathway when compared to DMSO, 11a, and Romidepsin, respectively (Fig. 6I). The examination of all genes in the MYC pathway identified the most differentially expressed genes in the combination group, which were verified by qRT-PCR (Fig. 6J, K) and immunoblotting (Fig. 6E). However, the comparisons of 11a vs DMSO and Romidepsin vs DMSO did not show significant inhibition of the MYC pathway by 11a or Romidepsin alone (Fig. 6L). The suppression of the MYC pathway only occurred in the combination group. This case represents synergy mode-2, termed “de novo” effect, which is unique to the combination (Fig. 6L). We also observed the “helper” mode in which Romidepsin did not inhibit the oxidative phosphorylation pathway by itself but strengthened the inhibition by 11a (Fig. 6L). We observed more examples of these synergy modes in the Hallmark collection (supplementary Fig. S12B). In summary, we have revealed how 11a and Romidepsin synergize to suppress cervical cancer, providing mechanistic insights into targeting NR2E3 in cancer.

## Discussion

### NR2E3 is a new tumor suppressor

We have concentrated our efforts on elucidating the tumor-suppressing roles of NR2E3. Our data demonstrate that NR2E3 enhances p53 transactivity in multiple cancer types, a function shared by other NR2E3 family members. Particularly noteworthy is NR2E3’s ability to restore wild-type p53 transactivity in the presence of mutated p53 (Fig. 1G). Given the prevalence of p53 mutations in cancer [41], leveraging NR2E3 to screen for such mutations holds promise. We recently constructed 50 nonsynonymous mutations of p53 that were detected in cancer patients. Preliminary data suggest that NR2E3 can partially restore the wild-type transactivities of certain p53 hotspot mutations. Furthermore, 25% of high-grade uterine endometrial cancer have frequent p53 mutations [42], in which activating NR2E3 may rescue wild-type p53 functions of a certain group of p53 mutations as done in Fig. 5B. Our study will offer a potential strategy to rectify mutated p53 by activating NR2E3 in cancer cells.

The increasing availability of databases highlighting the connections between NR2E3 SNVs and various solid tumors (Fig. 2A) underscores the importance of further exploring the roles of these SNVs in cancer. Our utilization of the p53 reporter assay has provided a straightforward and robust method for categorizing NR2E3 SNVs with significant implications in cancer (Fig. 2B−D). Leveraging this approach, we have elucidated that the cancer-associated NR2E3 R97H mutation contributes to tumorigenesis by failing to activate p53 (Fig. 3). In line with this, we recently constructed all of the five NR2E3 nonsynonymous mutations detected in multiple myeloma patients, one of which came to be a loss-of-function mutation.

Our observations suggest a potential role for NR2E3 in cancer predisposition, as all reported NR2E3 SNVs have been identified as germline mutations. Furthermore, these cancer-associated NR2E3 mutations often co-occur with mutations in various tumor-suppressing or tumor-promoting genes. For example, in a case of multiple myeloma, both NR2E3^E387K^ and NRAS^Q61R^ mutations were identified. Interestingly, our previous studies demonstrated that NRAS^Q61R^ alone was insufficient to induce multiple myeloma in germinal center B cells of wild-type C57BL/6 mice, but it was effective in C57BL/6 mice carrying the Vĸ-MYC insertion [43]. Given that NR2E3 has been shown to repress the MYC pathway, it is plausible that NR2E3^E387K^ may synergize with NRAS^Q61R^ to promote the development of multiple myeloma. This highlights the potential interplay between NR2E3 mutations and other genetic alterations in cancer pathogenesis. Currently, *Nr2e3*-Knock out and -Knock in mouse strains are available for in vivo evaluating NR2E3’s roles in cancer predisposition by cross-breeding with co-occurring gene mutations.

In contrast, the short isoform of NR2E3 behaves dominantly negatively to the full-length isoform, exhibiting tumor-promoting effects instead (Fig. 1D,E). High ratio of short vs FL correlates to inferior overall and disease-free survival in cancer patients (supplementary Fig. S9C, D). The balance between the short and full-length isoforms of NR2E3 is determined by alternative splicing that has been frequently dysregulated during tumorigenesis [44–46]. Targeting the alternative splicing machinery to generate more full-length isoform may change the cell fate of cancer cells as well as the retinal photoreceptors in enhanced *S*-cone syndrome. Additionally, this highlights the importance of considering alternative splicing in the study of NR2E3, particularly in transgenic mouse models where only one protein isoform is typically produced.

The transcriptomic studies provide valuable insight into the multifaceted tumor-suppressing roles of NR2E3, beyond its activation of the p53 pathway. By regulating pathways such as IFN-α, MYC, E2F, and ATP production, NR2E3 emerges as a versatile tumor suppressor with broad implications in cancer biology. Additionally, the observation of the dysplastic appearance and proliferative response observed in human retinal degenerations associated with mutated NR2E3 [12] opens up intriguing avenues for further research. Indeed, NR2E3 warrants comprehensive exploration in the context of cancer studies.

### NR2E3 is a new molecular vulnerability of cancer targeted by its agonist 11a

The observation that high expression levels of NR2E3 correlate with superior prognosis in various cancer types raises the question of how to activate NR2E3 for therapeutic benefit. To achieve this goal, Adeno-associated virus (AAV), which was approved as the gene therapy carrier for the treatment of lipoprotein lipase deficiency (Glybera) in 2012 and for the treatment of inherited vision loss (Luxturna) in 2017 [47, 48], can be used to deliver the wild-type full-length NR2E3 into cancer cells. Our efforts to study the functions of NR2E3 will shed light on the application of NR2E3 in gene therapy for cancer as well as for enhanced *S*-cone syndrome, one type of inherited vision loss.

Alternatively, our studies have shown that small-molecule 11a preferentially stimulates the NR2E3-mediated p53 transactivation, with the transcriptomic profile of 11a-treated cells closely resembling that of NR2E3-overexpressing cells (Fig. 4). We also show that 11a is able to penetrate uterine endometrial cancer mass in the explant culture, which is an important parameter of evaluating a drug candidate (Fig. 5A, B). Additionally, we have previously reported the wide-spectrum anti-cancer effects of 11a across the NCI-60 cancer cell panel [21]. Therefore, 11a emerges as a promising drug candidate for therapeutically activating NR2E3 and p53. Leveraging drug repurposing screens of FDA-approved anti-cancer drugs, we have established 11a-centered combinatorial treatments that effectively kill cervical cancer and retinoblastoma cells (Fig. 5). This approach holds great potential for identifying additional NR2E3-targeted combinatorial treatments for various cancer types.

The frequency of NR2E3 mutation in cancer is very low, and 11a will stimulate the wild-type of NR2E3 to activate p53 in cancer cells in most cases. However, the ability of 11a to stimulate NR2E3 mutations to activate p53 transactivity can be tested. We will evaluate the abilities of cancer-associated NR2E3 mutations to activate the p53 reporter on their own (Fig. 2C). Then we will assess if 11a can promote the NR2E3 mutations to activate the p53 reporter (Fig. 4C). Furthermore, the short isoform of NR2E3 lacks 43 amino acids in its ligand binding domain, which may damage the binding of 11a. Therefore, the examination of 11a’s binding to the short isoform will help to evaluate if 11a is still able to reverse the function of the short isoform.

The following mechanistic studies unveil that 11a suppresses ABCB1, a major contributor to resistance against numerous anti-cancer drugs, including Romidepsin [35, 49, 50]. Drug resistance stands as a primary cause of cancer relapse and mortality. By reducing ABCB1 expression, the efflux of anti-cancer drugs from cancer cells is diminished, thereby maintaining higher intracellular drug concentrations [51, 52]. In our study, 11a counteracted the Romidepsin-induced up-regulation of ABCB1 (supplementary Fig. S12A), highlighting another advantage of 11a in combinatorial therapies. Hence, further investigations into 11a, including pharmacokinetics and pharmacodynamics studies in preclinical models, are warranted.

## Supplementary information

supplementary information

supplementary figures

Full and uncropped Western blot images

## Data Availability

The RNA sequencing data were deposited to NCBI Sequence Read Archive (SRA) with the accession number PRJNA1192700. The rest datasets generated and/or analyzed during the current study are available from the corresponding author on reasonable request.
