# Supplementary material for: Orphan nuclear receptor NR2E3 is a new molecular vulnerability in solid tumors by activating p53
Source: Cell Death Dis. 2025 Jan 14;16(1):15. doi: 10.1038/s41419-025-07337-1 (PMC11733144; doi:10.1038/s41419-025-07337-1)
Supplement: Supplementary file 1 — supplementary information [file 41419_2025_7337_MOESM1_ESM.docx]

**Orphan nuclear receptor *NR2E3* is a new molecular vulnerability in solid tumors by activating p53**

Yidan Wang^1^, Todd Kroll^2,3^, Linhui Hao^4^, Zhi Wen^5^*

^1^McArdle Laboratory for Cancer Research, University of Wisconsin-Madison, Madison, WI 53705, USA.

^2^Department of Pathology, Marshfield Medical Center, Marshfield Clinic Health System, Marshfield, WI 54449, USA.

^3^Department of Pathology and Laboratory Medicine, Endeavor Northshore Health System, Evanston, IL 60201, USA.

^4^Institute of Molecular Virology, University of Wisconsin-Madison, Madison, WI 53705, USA.

^5^Center for Precision Medicine Research, Marshfield Clinic Research Institute, Marshfield Clinic Health System, Marshfield, WI 54449, USA.

*Corresponding author: Dr. Zhi Wen, e-mail: [wen.zhi@marshfieldclinic.org](mailto:wen.zhi@marshfieldclinic.org)

**Running title:** Activating NR2E3 suppresses cancer

**Key words:** Orphan nuclear receptor *NR2E3*, tumor suppressor p53, loss-of-function mutations, small-molecule NR2E3 agonist, anti-cancer drug repurposing screen

**Materials and Methods:**

**Plasmids and cell lines.** Human NR2E3 full-length, shRNAs against NR2E3, p53-C135Y, p53RE-SEAP, p53RE-FLuc, and SV40-RLuc were described [1]. All NR2E3 and p53 mutations were achieved with QuickChange II XL site-directed mutagenesis kit (Agilent, cat# 200521). NR2E3 truncates were made by PCR. All constructs were verified by Sanger sequencing.

Y79, MCF7 and HeLa cells were purchased from ATCC. *p53^-/-^* and *p53^+/+^* HCT116 cells were kindly provided by Dr. B. Vogelstein, John Hopkins University, H1299 cells by Dr. W. Sugden, University of Wisconsin-Madison, and RKO cells by Dr. P. Lambert, University of Wisconsin-Madison. FBS (GIBCO, cat# 16000069) was heat-inactivated at 56^o^C for 30 minutes and added to a final 10% concentration into DMEM (GIBCO, cat# 11-140-050) for HeLa, H1299, RKO and MCF7 cells, and McCoys’5A (Hyclone, cat# SH30200.FS) for HCT116 cells, and to a final 20% concentration into DMEM for Y79 cells. All the cell lines were cultured at 37^o^C in cell incubators filled with 5% CO_2_ and passaged every two or three days.

**Luciferases and SEAP reporter assay.** Luciferases/SEAP reporter assays were performed using 96-well plates and reverse transfection (RT). For each well, 0.01 µg of p53-responsive reporter plasmids, 0.005 µg of pSV40-RLuc plasmid, and the indicated amounts of NR2E3^WT^, NR2E3^MUT^, p53^WT^, p53^MUT^, or other plasmids were added to a total of 0.085 µg of DNA (balanced with empty vector) in 5 µL of Opti-MEM. 0.17 µL of TransIT-LT1 (Mirus, cat# MIR2305) was diluted with 9 µL of Opti-MEM, mixed with the DNA as described above, transferred to the microplate, and incubated for 30 min at room temperature. A total of 1.2 x 10^4^ cells in 100 µL of medium were then added per well. Two days after transfection, cells were processed using a Dual-Glo Luciferase assay (Promega, cat# E2940) and BMG Lumistar omega plate reader. SEAP (secreted alkaline phosphatase) activity was measured with Phospha-Light™ assay system (Pierce, cat#T1015). The relative FLuc or SEAP reads were calculated by dividing FLuc or SEAP reads by RLuc reads.

**Repurposing screens of FDA-approved anti-cancer drug library.** The AOD X library was kindly provided by Developmental Therapeutics Program, Division of Cancer Treatment and Diagnosis, National Cancer Institute. This library contains 166 FDA-approved anti-cancer drugs at 10 mM in 20 µL DMSO. These drugs were re-plated into 384-well Echo-compatible source plates (Beckman-Coulter, cat# C74290). Echo Plate reformat software was used to set up the cross-titration matrix of drugs. Drug combinations were spotted into white TC-treated clear bottom 384-well assay plates (Corning, cat# 3765) using the Beckman-coulter Echo 650 acoustic liquid handler. All assay points were backfilled with DMSO to a final volume of 50 nL per well. These plates will provide four final concentrations of the drugs as 5 µM, 300 nM, 20 nM and 1 nM when mixed with 50 µL of cell suspensions. The assay plates were sealed with sterile plate seals and stored at -20^o^C until needed.

50 µL 0.8x10^5^/mL HeLa cells or 3x10^5^/mL Y79 cells were seeded to each well of the assay plates by BioTek MultiFlo microplate dispenser with 5-µL stainless steel cassettes. The final concentration of 11a was 1.5 µM for HeLa cells and 4 µM for Y79 cells, respectively. After brief centrifugation at 200x g, the cells were cultured at 5% CO_2_, 37^o^C for two days. 20 µL CellTiter-Glo cell viability substrate (Promega, cat# G7573) were added per well with BioTek MultiFlo microplate dispenser with 5-µL plastic cassettes and incubated at room temperature for 10 minutes on a microplate shaker (USA Scientific, cat# 7402-4000). The luminescence was measured with BMG LUMIstar Omega plate reader. The cell viability in the DMSO control in each plate was normalized to 1. The normalized cell viabilities in the presence of 11a were plotted against those in the absence of 11a for each assayed concentration of the AOD X library. The outliers were selected by naked eyes.

**ZIP drug synergy scoring assay.** Romidepsin, Bortezomib and Carfilzomib (10 mM in DMSO) were purchased from TargetMol (Wellesley Hills, MA). 11a was synthesized by Pharmabridge Inc. (Doylestown, PA) and dissolved to 10 mM in ethanol alcohol and then DMSO [2].

The drugs were sequentially diluted in Opti-MEM medium (GIBCO, cat# 31985070) in 8-stripe PCR tubes. 5 µL each diluted drug was aliquoted with 8-channel P20 pipetman (Gilson, cat# F144070) to each well in a 96-well plate. 100 µL 1.2x10^5^/mL HeLa cells or 3x10^5^/mL Y79 cells were seeded to each well with 8-channel P200 pipetman (Gilson, cat# F144072). The cells were cultured at 5% CO_2_, 37^o^C for two days. 20 µL CellTiter-Glo cell viability substrate (Promega, cat# G7573) were added per well with DISTRIMAN Repetitive Pipette (Gilson, cat# F164001) and incubated at room temperature for 10 minutes on the microplate shaker (USA Scientific, cat# 7402-4000). The luminescence was measured with BMG LUMIstar Omega plate reader.

The cell viability in the DMSO control was normalized to 100%. ZIP synergy score was used to evaluate the synergy, following the instructions at [https://synergyfinder.fimm.fi](https://synergyfinder.fimm.fi/) [3]. “ZIP Average Score > 10” suggests a synergy between drugs; “ZIP Average Score < -10” suggests an antagonism between drugs; “ZIP Average Score between -10 and 10” suggests an additive effect between drugs.

**Treatment of patient cancer specimens with 11a in explant culture.** Uterine endometrial cancer mass was removed from patients through surgery, opened under the sterile settings in Pathology lab, allocated to DMEM + 10% FBS, 1x Penicillin + Streptomycin in a 10-cm x 2.5-cm Petri dish after clinic applications, and immediately transported in a biosafety container to Research lab. Tumor mass was sliced into < 3-mm masses in medium with sterile disposable scalpel. The small tumor masses were sucked up by 1-mL tip and transferred into a 6-well TC-treated plate containing 4 mL medium per well. DMSO or 4 µM 11a was used to treat tumor mass at 5% CO_2_, 37^o^C for 24 hours. The small tumor masses were then sucked up by 1-mL tip and transferred into 250 µL RIPA buffer (Pierce, cat# 89900) containing 1x Halt protease inhibitor cocktail (Thermo, cat# 78430) and 1x SDS sample buffer in a 1.5-mL microcentrifuge tube. These small tumor masses were sonicated with Branson SFX250 sonicator for three cycles of 20% power on for 15 seconds and power off for 60 seconds on ice. After heated at 100^o^C for 10 minutes, the cell lysates were centrifuged at 10,000x g, 4^o^C for 5 minutes. The supernatant was collected for immunoblotting assay.

The appendix of “Samples of Unlinked Fresh Tissue Excess” to the approved IRB protocol was approved by the Institutional Review Board of Marshfield Clinic Research Institute in September 2023. The IRB protocol number is IRB-21-88 with MCR Code WEN10221. Three samples were collected between November 2023 and June 2024 with Patient consent form waived.

**Bulk RNA sequencing.** The assays were supported by UW-Madison. To study NR2E3 wild-type and R97H, 2 µg of each plasmid (supplemented with empty vector to a total 4 µg) were transfected into HeLa cells with 8 µL TransIT-LT1 (Mirus, cat# MIR2300) 18 hours after 1x10^6^ HeLa cells were seeded in 6-cm dishes. 1 day after transfection, the medium was replaced with fresh medium. 2 days after transfection, RNA was extracted with RNeasy Mini kit (Qiagen, cat# 74106). RNA quantity and quality assessment was done with Nanodrop and Bioanalyzer 2100, library construction of mRNA was completed with NEBNext Ultra II RNA Library Prep kit for Illumina (NEB, cat# E7765) following the manual instruction, quantity and size distribution of the library was evaluated with Qubit and Bioanalyzer 2100, and libraries were pooled at equal molar concentrations, loaded on a NovaSeq 6000 S4 flow cell, and run on a NovaSeq 6000 sequencer. Each sample was sequenced at 20M reads with 2x150bp paired end.

To study the drug combination, 150 nM 11a and/or 5 nM Romidepsin were added to HeLa cells 1 day after 1x10^6^ HeLa cells were seeded in 6-cm dishes. RNA was extracted 1 day after drug treatments. RNA quantity and quality assessment was done with Nanodrop and Bioanalyzer 2100, library construction of mRNA was down with BGI in-house kit, quantity and size distribution of the library was done with Qubit and Bioanalyzer 2100, equal amount of each library was pooled and loaded to DNBSEQ-T7 PLATFORM, and each sample was sequenced at 20M reads with 2x150bp paired end.

The fastq files were aligned to hg38 reference genome and counted with STAR app and the differential expression between samples were acquired with DESeq2 app of RNA-Seq pipeline at [www.basepairtech.com](http://www.basepairtech.com). GSEA 4.1.0 application was used to conduct the pathway analysis with the v2023.1 human gene sets [4]. NIH David Function was used for pathway analysis [5]. All the fastq data were submitted to NCBI Sequence Read Archive (SRA) with the accession number PRJNA1192700.

**Chromatin Immunoprecipitation (ChIP).** The assays were done at UW-Madison. HeLa cells were co-transfected with 1.2 µg p53 plasmid and 2.4 µg NR2E3 WT plasmid/empty vector in 10-cm dishes. Two days later, the cells were processed following the protocol which included the sequences of the PCR primers of p53BS1 and p53BS2 on p21 promoter and p53BS on MDM2 intron. Primer sequences were provided separately. Detailed ChIP protocol is following:

1. Two days after transfection, HeLa cells were washed twice with 10 mL PBS

2. Add 270 µL formaldehyde (from 37% stock, final is 1%) into 10 mL PBS per 10-cm dish, mix immediately and incubate at room temperature (RT) for 30 minutes

3. Add 1.25 mL 1.25M glycine and incubate at RT for 5 minutes with gentle shaking

4. Place the dishes on ice and wash once with 10 mL cold PBS

5. Harvest the cells with scrapers into 10 mL cold PBS and centrifuge at 1,500x rpm at 4^o^C for 5 minutes in a Beckman-Coulter GPR centrifuge

6. Wash cell pellets with 10 mL buffer I (0.25% TritonX100, 10mM EDTA, 0.5 mM EGTA, 10 mM HEPES) and II (200mM NaCl, 1mM EDTA, 0.5mM EGTA, 10mM HEPES, pH 6.5) sequentially

7. Resuspend the cells in 1 mL lysis buffer (1% SDS, 10mM EDTA, 50mM Tris, pH 8.1) with protease inhibitors

8. Sonicate the cells with microtip (10 seconds at 50% power input with 2 minutes rest on ice for 8 cycles. Do not get too cold, or the SDS will precipitate in the buffer) using a Branson Digital Sonifier 450 sonicator

9. Transfer the cell lysates into 1.5-mL microtubes and centrifuge at 15,000x rpm at 10^o^C for 15 minutes

10. Dilute the supernatant 10 fold with cold dilution buffer (1% TritonX100, 2mM EDTA, 150mM NaCl, 20mM Tris, pH 8.1) with protease inhibitor cocktails in 15-mL tubes on ice. Leave 100 µL aliquot as input control

11. Incubate the diluted chromatin solution with 3 µL anti-acetylated p53 antibody (Upstate, cat# 06-758) on a nutator in cold room for 12 to 16 hours

12. Add 50 µL 50% slurry of protein A-sepharose 4B beads (washed in TE pH 8.0; blocked with ssDNA) and incubate on the nutator in cold room for 1 hour

13. Centrifuge at 1,500x rpm at 4 ^o^C for 2 minutes to pellet the beads and discard the supernatant

14. Resuspend the beads in 1 mL TSE+150mM NaCl buffer (0.1% SDS, 1% TritonX-100, 2mM EDTA, 20mM Tris, 150mM NaCl, pH 8.1) and wash the beads with a Fisher Genie 2 Vortex at setting 1 in cold room for 10 minutes.

15. Spin and wash the beads again with 1 mL TSE+500mM NaCl buffer (0.1% SDS, 1% TritonX-100, 2mM EDTA, 20mM Tris, 500mM NaCl, pH 8.1) and then with buffer III (0.25M LiCl, 1% NP-40, 1% deoxycholate, 1mM EDTA, 10mM Tris, pH 8.1)

16. Wash beads twice with 1 mL TE buffer and elute the immune complexes with 300 µL 1% SDS, 0.1M NaHCO_3_ with Fisher Genie 2 Vortex at setting 6 at RT for 30 minutes

17. Incubate the elutions and the aliquots (the input control) at 65^o^C for 4 to 6 hours

18. Purify DNA with PCR purification kit (Qiagen, cat# 28104)

19. Use GoTaq PCR kit (Promega, cat# M7660) to amplify interested fragments (50 ^o^C, 2 minutes; 95 ^o^C, 10 minutes; 29 cycles of 95 ^o^C, 15 seconds; 55 ^o^C, 30 seconds and 72 ^o^C, 30 seconds; 72 ^o^C, 7 minutes; and 4 ^o^C forever) for electrophoresis in 2% agarose gel containing ethidium bromide staining with a thin comb for images

20. Use TaqMan™ Fast Advanced Master Mix (Applied Biosystems, cat# 4444557) and ABI 7900HT device to quantify the ChIP products

**Gel shift assay (EMSA).** The assays were done at UW-Madison with the certificate for radioactive experiments using isotope ^32^P. HeLa cells were co-transfected with both 0.6 µg p53 plasmid and 0.6/1.2 µg NR2E3 WT, 1.2 µg R76W/R97H plasmids or empty vector in 6-cm dishes, respectively. Two days later, nuclear extracts were harvested [1]. The sequence of p53-binding consensus and the protocol were provided separately. Detailed EMSA protocol is following:

1. Anneal DNA primers:

10x NEB buffer 2 10 µL

Primer 1 (100uM, 1.1 µg/µL) 1 µL

Primer 2 (100uM, 1.1 µg/µL) 1 µL

H_2_O 38 µL

PCR program: 94 ^o^C, 2 minutes; 65 ^o^C, 10 minutes; 37 ^o^C, 10 minutes; and 4 ^o^C forever

2. Label probe with ^32^P-dCTP:

5xbuffer 3 µL

Annealed Probe 2.5 µL

Klenow (exo-) DNA polymerase (5u/µL) 1 µL

ddH_2_O 36.5 µL

^32^P dCTP 5 µL

Incubate at 37 ^o^C for 2-10 minutes and add 2 µL stop solution

Pre-spin G-25 column for 1 minute at 735x g with lip loosen

Load 50 µL reaction product into the center of slope and spin at 735x g for 2 minutes

Count flow-through with program 27

3. Prepare 4% nature PAGE Gel:

Treat all parts in 0.1% NaOH overnight. Rinse all parts with tap water, clean with Windex followed by 70% ethanol, rinse with Millipore H_2_O, and then dry. Wipe all parts with DNA/RNase removal reagent before use

50 mL 4% Gel:

30% Acrylamide:Bis (29:1) 6.67mL

10x TBE 1.25mL

10%APS 0.33mL

TEMED 40µL

ddH_2_O 41.7mL

Rinse wells and pre-Run gel in 0.5x TBE buffer at 100V, 1-2 hours

4. 2x mother mixture:

40% Glycerol and 2mM EDTA in 80mM Tris, pH 7.5 5 µL

KCL (1M) 1 µL

MgCl_2_ (100mM) 1 µL

BSA (10mg/mL) 1 µL

DTT (20mM) 1 µL

Salmon sperm DNA (1mg/mL) 2 µL

Total Volume 11µL

5. Reaction:

2x Mother mixture 11µL

Nuclear extract 4µL

ddH_2_O 3µL

Block at RT for 5 minutes

*(For probe competition, add 50x cold probe and incubate at RT for another 5 minutes)*

^32^P-probe 2µL

Incubate at RT for 15-30 minutes

Load 18 µL or all into gel

Run 2.5 hours at 150V at RT until the upper dye approaches 8 cm away from gel bottom

6. Dry gel:

Cover the gel with one piece of Whatman 3M paper and peer the gel off the plate

Stack the first Whatman paper on a second paper

Cover the other face of gel with Sara membrane and avoid bubbles

Transfer the gel within a radiation-protective container into gel dryer

Add ethanol and dry ice into the tank of gel dryer

Put the gel to the dryer with the paper facing to the bottom and cover the gel with dryer pad

Dry gel at 70 ^o^C for 1 hour

7. Exposure:

Pre-bleach the phosphor-storage plate

Replace the second Whatman paper with a third paper

Put the dried gel in the plate and cover for overnight

Scan the plate with Typhoon machine at 100-200 mp

**Immunoblotting.** The main protocol was previous published [1]. Of note, acetylated p53 at K373+K382, acetylated p53 at K319 and acetylated p53 at K386 were processed for incubation with the primary antibodies in Fish Serum blocking buffer (Thermo, cat# 37527) at RT for two hours. The anti-NR2E3 antibody (Santa Cruz, cat# sc-374513) preferentially detects the short isoform with high sensitivity, but it is not similar sensitive for the full-length (FL). The FL must be processed for incubation with the primary antibody in 5% non-fat milk at 4^o^C overnight. The other antigens were often processed with primary antibodies in 5% non-fat milk at 4^o^C overnight, and occasionally for incubation with primary antibodies in Fish Serum blocking buffer at RT for two hours.

**Bioinformatics and statistics.**

GEPIA tool [6] at <http://gepia.cancer-pku.cn/detail.php> was used to analyze the survival data in TCGA database. The Group cutoff was set as: High is >60% and low is <40%. NR2E3 RNA levels were normalized by β-actin. *p* values were calculated by Logrank test.

NR2E3 gene expression profile in human tissues was obtained and sorted using GTEx online tool at <https://gtexportal.org/home/gene/NR2E3#geneExpression> [7].

NCBI ClinVar tool at <https://www.ncbi.nlm.nih.gov/clinvar/?term=NR2E3%5Bgene%5D> was used to review SNVs of NR2E3.

The association study of NR2E3 mutations and cancer was done between TCGA and “All *of* Us” databases. “All *of* Us” database allows to stratify the population of > 182,000 cases with comprehensive information into sub-groups based on race, age and gender. Mutations that change NR2E3 protein sequence were collected in both databases. Chi-square test with two tails and 1 degree of freedom was used to calculate *p* values only of the comparisons with minimal case number of 5. Either Woolf logit test (> 99,000 cases) or Baptista-Pike (< 99,000 cases) test was used to calculate OR values with 95% confidence interval.

Mean + SD was shown in all histograms. The samples size was n > 3. *p* values were calculated by one-way Anova test with Bonferroni post-hoc test, Student’s t-test with two tails, and Logrank test.

Data visualization technique with Histograms was used to test if the data meet the assumptions of normal distribution. Anova test was used to test the variance between groups and within each group.

**Immunofluorescence, Co-Immunoprecipitation, RNA interference, Cycloheximide treatment, Reverse transcription followed by Real time-PCR, cell transfection, and cell apoptosis assay** were previous published [1, 8].

**Mutation primers:**

| Mutation | Primer sequence | |
| --- | --- | --- |
| NR2E3-R76W | FWD | CAA GAG GAG CGT AtG GCG GAG GCT CAT C |
|  | REV | GAT GAG CCT CCG CCa TAC GCT CCT CTT G |
| NR2E3-G88V | FWD | GCC AGG TGG GGG CAG tGA TGT GCC CCG |
|  | REV | CGG GGC ACA TCa CTG CCC CCA CCT GGC |
| NR2E3-R97H | FWD | GAC AAG GCC CAC CaC AAC CAG TGC CAG |
|  | REV | CTG GCA CTG GTT GtG GTG GGC CTT GTC |
| NR2E3-E121K | FWD | GCC GTG CAG AAC aAG CGC CAG CCG CG |
|  | REV | CGC GGC TGG CGC TtG TTC TGC ACG GC |
| NR2E3-V302I | FWD | CAT GGA GAC GCG TaT CCT GCA GGA AAC |
|  | REV | GTT TCC TGC AGG AtA CGC GTC TCC ATG |
| NR2E3-M407K | FWD | CTC CTT TGT GAT AaG TTC AAA AAC TGA ATT C |
|  | REV | GAA TTC AGT TTT TGA ACt TAT CAC AAA GGA G |
| HA-NR2E3 short | FWD | GTT GTG TTA CCA TGT ATC CAT ATG ACG TCC CAG ACT ATG CCA TGG AGA CCA GAC CAA CAG CTC TG |
|  | REV | GTT GTG AGC TCT CAC CTC ACG GGC TGG CTG GGG TG |
| HA-NR2E3-DBD | FWD | GTT GTG TTA CCA TGT ATC CAT ATG ACG TCC CAG ACT ATG CCA TGG AGA CCA GAC CAA CAG CTC TG |
|  | REV | GTT GTG AGC TCT CAG GAC TCA GTG TTG GAC TCC ATG CTG |
| HA-NR2E3-LBD | FWD | GTT GTG TTA CCA TGT ATC CAT ATG ACG TCC CAG ACT ATG CCA TGC ATG AGA CCT CGG CTC GCC TAC TCT TCA TGG |
|  | REV | GTT GTG AGC TCT CAG TTT TTG AAC ATA TCA CAA AGG AG |
| p53-L25-26A | FWD | CAG ACC TAT GGA AAg cAg cTC CTG AAA ACA AC |
|  | REV | GTT GTT TTC AGG Agc Tgc TTT CCA TAG GTC TG |
| p53-R249S | FWD | CAT GAA CCG GAG tCC CAT CCT CAC |
|  | REV | GTG AGG ATG GGa CTC CGG TTC ATG |
| p53-R273H | FWD | GAA CAG CTT TGA GGT GCa TGT TTG TGC CTG TCC TG |
|  | REV | CAG GAC AGG CAC AAA Cat GCA CCT CAA AGC TGT TC |
| p53-R306A | FWD | CAG GGA GCA CTA AGg cAG CAC TGC CCA ACA ACA C |
|  | REV | GTG TTG TTG GGC AGT GCT gcC TTA GTG CTC CCT G |

**ChIP-PCR and EMSA primers:**

| p53BS1 on p21 [9] | FWD | GTG GCT CTG ATT GGC TTT CTG |
| --- | --- | --- |
|  | REV | CTG AAA ACA GGC AGC CCA AG |
| p53BS2 on p21 [9] | FWD | CCG AGG TCA GCT GCG TTA GAG |
|  | REV | GCA GAG GAT GGA TTG TTC ATC |
| p53BS on MDM2 [10] | FWD | TGG GCA GGT TGA CTC AGC TTT |
|  | REV | CCA GCT GGA GAC AAG TCA GGA |
| p53 EMSA probe | FWD | GAG TAC AGA ACA TGT CTA AGC ATG CTG GGG ACT |
|  | REV | GTG AGT CCC CAG CAT GCT TAG ACA TGT TCT GTA |

**qRT-PCR primers:**

| Gene | Primer sequence | |
| --- | --- | --- |
| p53BS1 on p21 | FWD | GCTGTGGCTCTGATTGGCTTT |
|  | Probe | FAM-TGTCCCAAC |
|  | REV | TTAGAGGTCTCCTGTCTCCTA |
| p53BS2 on p21 | FWD | ACAGCAGAGGAGAAAGAAGCC |
|  | Probe | FAM-TGCGTTAGA |
|  | REV | TCTCAGGCTCAGAGTCTG |
| p53BS on MDM2 | FWD | TGGGCAGGTTGACTCAGCTTT |
|  | Probe | FAM-GTTCAGACACGTTCCGAAACTGCAGT |
|  | REV | CCAGCTGGAGACAAGTCAGGA |
| UBE2L6 | FWD | CCACGGATGAGTCACAATCT |
|  | REV | CCCAGGAACTGGCAATCTAA |
| EGR1 | FWD | CTCTACTGGAGTGGAAGGTCTA |
|  | REV | GAACTTGGACATGGCTGTTTC |
| DHRS2 | FWD | GGTCTCTTCCATTGCAGCTTAT |
|  | REV | CTCCAATGCCAGTGTTCTAGTG |
| IFI6 | FWD | GCTAGAGTGCAGTGGCTATT |
|  | REV | GTAATCCTACTTGGGAGGTTGAG |
| IFI27 | FWD | CTGTCATTGCGAGGTTCTACT |
|  | REV | ATTTGGGATAGTTGGCTCCTC |
| OAS1 | FWD | CAGTTGACTGGCGGCTATAA |
|  | REV | TGTGAAGCAGGTGGAGAAC |
| OAS3 | FWD | GATGAGGGAGTGGGTCTATCT |
|  | REV | TGGAGAGTCAGGCTGTCTAA |
| ABCB1 | FWD | CTTCATCGAGTCACTGCCTAAT |
|  | REV | TAACAAGGGCACGAGCTATG |
| CA9 | FWD | GCTGTCTCGCTTGGAAGAA |
|  | REV | TATTGGAAGTAGCGGCTGAAG |
| IGF1 | FWD | AACAAGCCCACAGGGTATG |
|  | REV | ACATCTCCAGCCTCCTTAGA |
| CHAC1 | FWD | GGAGGCTTCTCTTTCTCAGTC |
|  | REV | CACACCAACATGGTGCAATAA |
| UNC5B | FWD | CAAGGACAGTTACCACAACCT |
|  | REV | CTGCCACTCCAAATGTGATAGA |
| ATF3 | FWD | CAGTTCCAAAGTCACAGGAAGA |
|  | REV | CCTAGACACAACTCCTGACCTA |
| DDIT3 | FWD | AGGGAGAACCAGGAAACGGAAACA |
|  | REV | TCCTGCTTGAGCCGTTCATTCTCT |
| TXNIP | FWD | CACTCTCAGCCATAGCACTTT |
|  | REV | CATCTTCAGCCCACACTTTCT |
| TYMS | FWD | CAAATCTGAGGGAGCTGAGTAA |
|  | REV | GAACAAAGCGTGGACGAATG |
| HDGF | FWD | TGGTCTCTCTATGCCTCTCTAC |
|  | REV | ACGGTTCTCAGAGCTAAACTTC |
| CCNA2 | FWD | CTTCACCAGACCTACCTCAAAG |
|  | REV | GGTGGGTTGAGGAGAGAAAC |

| ACTB | FWD | CACTCTTCCAGCCTTCCTTC |
| --- | --- | --- |
|  | REV | GTACAGGTCTTTGCGGATGT |
| GAPDH | FWD | GCCTCAAGATCATCAGCAATGCCT |
|  | REV | TGTGGTCATGAGTCCTTCCACGAT |
| B2M | FWD | TGTGTCTGGGTTTCATCCATCCGA |
|  | REV | TCACACGGCAGGCATACTCATCTT |
| RPL38 | FWD | GCAGATACCTTTACACCCTGG |
|  | REV | CTGGTTCATTTCAGTTCCTTCAC |

**Antibodies:**

1:3,000 mouse anti-p53 antibody (Santa Cruz, cat# sc-126), 1:1,000 mouse anti-p21 antibody (Santa Cruz, cat# sc-6246), 1:1,000 mouse anti-DDIT3 antibody (Santa Cruz, cat# sc-7351), 1:1,000 mouse anti-ATF3 antibody (Santa Cruz, cat# sc-81189), 1:1,000 mouse anti-Puma antibody (Santa Cruz, cat# sc-374223), 1:500 mouse anti-NR2E3 antibody (Santa Cruz, cat# sc-374513), 1:1,000 mouse anti-GAPDH antibody (Santa Cruz, cat# sc-137179), 1:3,000 mouse anti-β-actin antibody (Santa Cruz, cat# sc-47778), 1:500 mouse anti-p300 antibody (Santa Cruz, cat# sc-584), 1:5,000 mouse anti-HA antibody (Sigma, cat# 11583816001).

1:2,000 rabbit anti-β-actin antibody (Santa Cruz, cat# sc-1616), 1:2,000 rabbit anti-GFP antibody (Santa Cruz, cat# SC-8334), 1:3,000 rabbit anti-p53 antibody (Proteintech, cat# 10442-1-AP), 1:1,000 rabbit anti-Acetyl-p53-K373+K382 antibody (Sigma, cat# 06-758), 1:1,000 rabbit anti-Acetyl-p53-K386 antibody (Sigma, cat# Sab4503020), 1:1,000 rabbit anti-Acetyl-p53-K319 (Sigma, cat# Sab4503014), 1:6,000 rabbit anti-HA antibody (Sigma, cat# H6908), 1:3,000 rabbit anti-PARP antibody (Cell signaling, cat# 9542L), 1:3,000 rabbit anti-CCNA2 antibody (Bethyl, cat# A305-253A)

1:10,000 LI-COR IRDye 800CW Goat anti-Rabbit IgG (LI-COR, cat# NC9401842), 1:10,000 IRDye 680LT Goat anti-Mouse IgG (LI-COR, cat# NC0046410), 1:10,000 Goat-anti-mouse antibody conjugated with HRP (Thermo, cat# 31430).

**Supplemental figure legends:**

**Figure S1: Gene-expression profiles of NR2E3.** (A) *NR2E3* is highly expressed in human urogenital systems (red font), thyroid, lung, *etc.*, besides in retinal photoreceptor cells. Data were extracted from GTEx Analysis Release V8 (dbGaP phs000424.v8.p2) at <https://gtexportal.org/home/gene/NR2E3#geneExpression> [7]. (B) *Nr2e3* expression in mouse urogenital system and respiratory system was repeatedly detected. Data were extracted from Mouse Genome Informatics at <https://www.informatics.jax.org/gxd/phenogrid/MGI:1346317> [11].

**Figure S2: Subfamily members of NR2E3 activate p53 transactivity.** 0.01 µg NR2E3 or its subfamily members NR2E1, NR2F1 and NR2F2 that are all orphan nuclear receptors were co-transfected with the p53RE-SEAP reporter and SV40-RLuc into HeLa cells (A) and *p53^+/+^* HCT116 cells (B) in 96-well plates. The relative SEAP activity in control was normalized to 1. Mean + SD shown in all the histograms. *p* value was calculated by one-way Anova test and Bonferroni post-hoc test (each family member vs NR2E3 vs Con). *: *p*<0.0167; **: *p*<0.0033; ***: *p*<0.00033.

**Figure S3: NR2E3 selectively rescued the wild-type transactivities of p53 mutations which partially reserve this transactivity, but not the other mutations which completely lose it in p53-null H1299 cells.** (A) The indicated p53 plasmids (0.008 µg) was co-transfected with the p53RE-FLuc reporter and SV40-RLuc, and with or without 0.01 µg NR2E3 plasmid into p53-null H1299 cells in 96-well plates. Two days later, the luminescence activity was measured. The relative FLuc activity in the p53^WT^+empty vector control was normalized to 1. Mean + SD shown. *p* value was calculated by Student’s t-test with two tails. **: *p*<0.01. ***Notes:*** L25-26A is a mutation at the p53 TAD1 domain. R306A is a mutation at the p53 NLS domain. C135Y is a dominant-negative mutation. R249S is a hot-spot mutation. R273H is a gain-of-function mutation. (B) Immunoblotting assay showed the protein levels of p53 mutations in p53-null H1299 cells two days after transfection with 0.2 µg of the indicated p53 mutations and 0.05 µg GFP and with or without 0.6 µg FL into 0.5 x 10^6^ p53-null H1299 cells in 6-well plates. *GFP*: transfection reference.

**Figure S4:** RNA levels of growth-inhibitory genes were measured by qRT-PCR in *p53^-/-^* H1299 cells two days after transfection with 4.0 µg FL or empty vector in 6-cm dishes. Mean + SD shown. *p* value was calculated by Student’s t-test with two tails. *: *p*<0.05; **: *p*<0.01; ***: *p*<0.001.

**Figure S5: Gene Set Enrichment Analysis (GSEA) of published RNA microarray data [12] suggests tumor inhibitory roles of NR2E3 in mouse.** Retinal tissues of age-matched wild-type (WT) mice, *NR2E3^-/-^* (KO) mice and *rd7/rd7* (*rd7*) mice at C57BL/6J strain were harvested at different time points after birth. RNA was extracted for RNA microarray studies. The 302 most differentially expressed genes that were listed in the supplemental data were re-analyzed by GSEA. (A) Summary of the NES and FDR values of enriched gene sets between *rd7* and WT mice and between KO and WT mice. NRL- and CRX-related gene sets were presented as internal positive controls of re-analysis because NR2E3 regulates and is regulated by NRL and CRX [13-15]. (B-C) Representative tumor-related gene sets enriched in KO mice and in *rd7* mice.

**Figure S6: Overview of Single Nucleotide Variants (SNV) of NR2E3.** The data were collected from ClinVar (<https://www.ncbi.nlm.nih.gov/clinvar/?term=NR2E3%5Bgene%5D>). (A) Classifications of NR2E3 SNVs. (B) Clinical involvements of NR2E3 SNVs. (C) Summary of six diseases-associated NR2E3 mutations. *red font*: Pathogenic. R97H was detected in 2 out of 512 uterine endometrial cancer cases in a TCGA cohort.

**Figure S7: NR2E3 SNVs differentially regulate the p53 reporter.** (A) 0.01 µg NR2E3 mutations were co-transfected with the p53RE-SEAP reporter and SV40-RLuc into in *p53^+/+^* HCT116 cells. Two days later, the luminescence activity was measured. The relative SEAP activity in the control was normalized to 1. (B) 0.01 µg NR2E3 mutations were co-transfected with the p53RE-SEAP reporter, SV40-RLuc and 0.008 µg p53^WT^ expression plasmid into in p53-null H1299 cells. Two days later, the luminescence activity was measured. The relative SEAP activity in the control was normalized to 1. Mean + SD shown in all the histograms. *p* value was calculated by one-way Anova test and Bonferroni post-hoc test (each mutation vs Con vs FL). *: *p*<0.0167; **: *p*<0.0033; ***: *p*<0.00033.

**Figure S8: Representative images of Flow Cytometry analysis in Figure 2-D.** (A) BD FacsCalibur device was used. (B) BD LSR-Fortessa device was used.

**Figure S9:** (A-B) High expression levels of NR2E3 were correlated to superior Disease-free cancer survival (A) and overall survival (B) of 33 cancer types. GEPIA tool [6] was used to analyze the data in TCGA database. The group cutoff: High is > 60%; and low is < 40%. RNA levels of NR2E3, FL and short were normalized to β-actin. *p* values were calculated by Logrank test. (C-D) Imbalance of NR2E3 isoforms showed that high FL levels were correlated to superior Disease-free cancer survival (C) and overall survival (D) of 33 cancer types. GEPIA tool [6] was used to analyze the data in TCGA database. The group cutoff: High is > 60%; and low is < 40%. RNA levels of FL were normalized to the short. *p* values were calculated by Logrank test.

**Figure S10: 11a activates p53-targeted genes in *p53^+/+^* HCT116 cells.** HCT116 cells were treated with the indicated concentrations of 11a for 24 hours. The cell lysates were processed for Immunoblotting assays with the indicated antibodies. Three biological repeats were conducted.

**Figure S11:** Gene Set Enrichment Analysis of published RNA microarray data [12] suggests the tumor suppressing roles of 11a. Retinal explants of wild-type C57BL/6J mice were cultured overnight in the presence or absence of 11a. RNA was extracted for RNA microarray studies. The 360 most differentially expressed genes that were listed in the supplemental data were re-analyzed with GSEA. Representative tumor-promoting gene set was enriched in DMSO group.

**Figure S12:** (A) 11a reverses the induction of ABCB1 by Romidepsin in HeLa cells. HeLa cells were treated with DMSO, 11a (150 nM), Romidepsin (5 nM) and the combo for 24 hours, respectively. Total RNA was extracted for qRT-PCR assay. Mean + SD shown. *p* value was calculated by one-way Anova test and Bonferroni post-hoc test (DMSO vs 11a vs Romidepsin vs Combo). *: *p*<0.0083; **: *p*<0.00167; ***: *p*<0.00017. (B) Summary of Hallmark GSEA data in the comparisons of 11a vs DMSO, Romidepsin vs DMSO, Combo vs DMSO, Combo vs 11a, and Combo vs Romidepsin. Not enriched: FDR > 0.25.

**Supplemental discussion**

Our previous study [2] used the DR2-reporter to test 11a based on the general assumption that any nuclear receptor may activate the DR2-reporter. However, the DR2 reporter has not been validated for NR2E3 study. More importantly, NR2E3 is a transcriptional co-activator of p53 [1]. Though it has been studied as a transcription factor in many other studies, NR2E3, being a transcriptional co-activator in our studies, does not have to bind to and activate its own targets as a transcription factor often does. Indeed, a transcriptional co-activator often relies on, forms a complex with, and activates its transcription factor which is p53 in our case [1]. Therefore, we used the p53-responsive reporter to test NR2E3 and its agonist 11a in the current study. Our results showed that 11a activates the p53 transactivities mainly through NR2E3 (Fig. 4B-E). Furthermore, 11a also activates, but in a lower degree, the family members of NR2E3 to stimulate p53 transactivities (Fig. 4B).

Of note, we have also provided new evidence in *p53^+/+^* HCT116 cells (Fig. S10) to improve upon our previously study [2] in which p53 protein level was not demonstrated in 11a-treated HCT116 cells. Our previous study also failed to detect endogenous NR2E3 in HCT116 cells with a low-sensitivity antibody against NR2E3. With recently available antibodies against NR2E3, we and others have detected endogenous NR2E3 in multiple tissues and cancer types of both human and mouse, including HCT116 cells (Fig. 3J, 4D-E, 5B, 6E, and S10).

Besides the 293T cells, in which p53 is repressed by SV40 T antigen, our previous study utilized MDA-MB-231, LM2, and MDA-MB-468 cell lines, which express mutated p53 R280K and R273H, respectively, to study 11a’s activation of wild-type p53. These cell line models were corrected in the current study. We also showed that NR2E3 does not activate p53 R273H (Fig. 1G and Fig. S3). Excitingly, we recently constructed 50 p53 mutations that were detected in multiple myeloma patients, and the preliminary data showed that NR2E3 is able to activate a subgroup of them, including some hotspot mutations (data not shown). In sum, we have been sincerely making progress in addressing these unsolved questions on NR2E3. Our and other results have shown that NR2E3 is emerging as a novel tumor suppressor [1, 2, 16-19].

**Supplemental references**

1 Wen Z, Pyeon D, Wang Y, Lambert P, Xu W, Ahlquist P. Orphan nuclear receptor PNR/NR2E3 stimulates p53 functions by enhancing p53 acetylation. *Mol Cell Biol* 2012; 32: 26-35.

2 Zhao Z, Wang L, Wen Z, Ayaz-Guner S, Wang Y, Ahlquist P *et al*. Systematic analyses of the cytotoxic effects of compound 11a, a putative synthetic agonist of photoreceptor-specific nuclear receptor (PNR), in cancer cell lines. *PLoS One* 2013; 8: e75198.

3 Ianevski A, Giri AK, Aittokallio T. SynergyFinder 3.0: an interactive analysis and consensus interpretation of multi-drug synergies across multiple samples. *Nucleic acids research* 2022; 50: W739-W743.

4 Mootha VK, Lindgren CM, Eriksson KF, Subramanian A, Sihag S, Lehar J *et al*. PGC-1alpha-responsive genes involved in oxidative phosphorylation are coordinately downregulated in human diabetes. *Nat Genet* 2003; 34: 267-273.

5 Sherman BT, Hao M, Qiu J, Jiao X, Baseler MW, Lane HC *et al*. DAVID: a web server for functional enrichment analysis and functional annotation of gene lists (2021 update). *Nucleic acids research* 2022; 50: W216-W221.

6 Tang Z, Li C, Kang B, Gao G, Li C, Zhang Z. GEPIA: a web server for cancer and normal gene expression profiling and interactive analyses. *Nucleic acids research* 2017; 45: W98-W102.

7 Consortium GT. Human genomics. The Genotype-Tissue Expression (GTEx) pilot analysis: multitissue gene regulation in humans. *Science* 2015; 348: 648-660.

8 Wen Z, Yun G, Hebert A, Kong G, Ranheim EA, Finn R *et al*. Nras Q61R/+ and Kras-/- cooperate to downregulate Rasgrp1 and promote lympho-myeloid leukemia in early T-cell precursors. *Blood* 2021; 137: 3259-3271.

9 Laptenko O, Beckerman R, Freulich E, Prives C. p53 binding to nucleosomes within the p21 promoter in vivo leads to nucleosome loss and transcriptional activation. *Proceedings of the National Academy of Sciences of the United States of America* 2011; 108: 10385-10390.

10 Ard PG, Chatterjee C, Kunjibettu S, Adside LR, Gralinski LE, McMahon SB. Transcriptional regulation of the mdm2 oncogene by p53 requires TRRAP acetyltransferase complexes. *Mol Cell Biol* 2002; 22: 5650-5661.

11 Baldarelli RM, Smith CM, Finger JH, Hayamizu TF, McCright IJ, Xu J *et al*. The mouse Gene Expression Database (GXD): 2021 update. *Nucleic acids research* 2021; 49: D924-D931.

12 Webber AL, Hodor P, Thut CJ, Vogt TF, Zhang T, Holder DJ *et al*. Dual role of Nr2e3 in photoreceptor development and maintenance. *Exp Eye Res* 2008; 87: 35-48.

13 Oh EC, Cheng H, Hao H, Jia L, Khan NW, Swaroop A. Rod differentiation factor NRL activates the expression of nuclear receptor NR2E3 to suppress the development of cone photoreceptors. *Brain Res* 2008; 1236: 16-29.

14 Hennig AK, Peng GH, Chen S. Regulation of photoreceptor gene expression by Crx-associated transcription factor network. *Brain Res* 2008; 1192: 114-133.

15 Peng GH, Chen S. Chromatin immunoprecipitation identifies photoreceptor transcription factor targets in mouse models of retinal degeneration: new findings and challenges. *Vis Neurosci* 2005; 22: 575-586.

16 Khanal T, Leung YK, Jiang W, Timchenko N, Ho SM, Kim K. NR2E3 is a key component in p53 activation by regulating a long noncoding RNA DINO in acute liver injuries. *Faseb J* 2019; 33: 8335-8348.

17 Park YY, Kim K, Kim SB, Hennessy BT, Kim SM, Park ES *et al*. Reconstruction of nuclear receptor network reveals that NR2E3 is a novel upstream regulator of ESR1 in breast cancer. *EMBO Mol Med* 2012; 4: 52-67.

18 Khanal T, Choi K, Leung YK, Wang J, Kim D, Janakiram V *et al*. Loss of NR2E3 represses AHR by LSD1 reprogramming, is associated with poor prognosis in liver cancer. *Sci Rep* 2017; 7: 10662.

19 Leung YK, Lee SG, Wang J, Guruvaiah P, Rusch NJ, Ho SM *et al*. The Loss of an Orphan Nuclear Receptor NR2E3 Augments Wnt/beta-catenin Signaling via Epigenetic Dysregulation that Enhances Sp1-beta catenin-p300 Interactions in Hepatocellular Carcinoma. *Adv Sci (Weinh)* 2024; 11: e2308539.
