# Supplementary material for: Orphan nuclear receptor NR2E3 is a new molecular vulnerability in solid tumors by activating p53
Source: Cell Death Dis. 2025 Jan 14;16(1):15. doi: 10.1038/s41419-025-07337-1 (PMC11733144; doi:10.1038/s41419-025-07337-1)
Supplement: Supplementary file 2 — supplementary figures [file 41419_2025_7337_MOESM2_ESM.pdf]

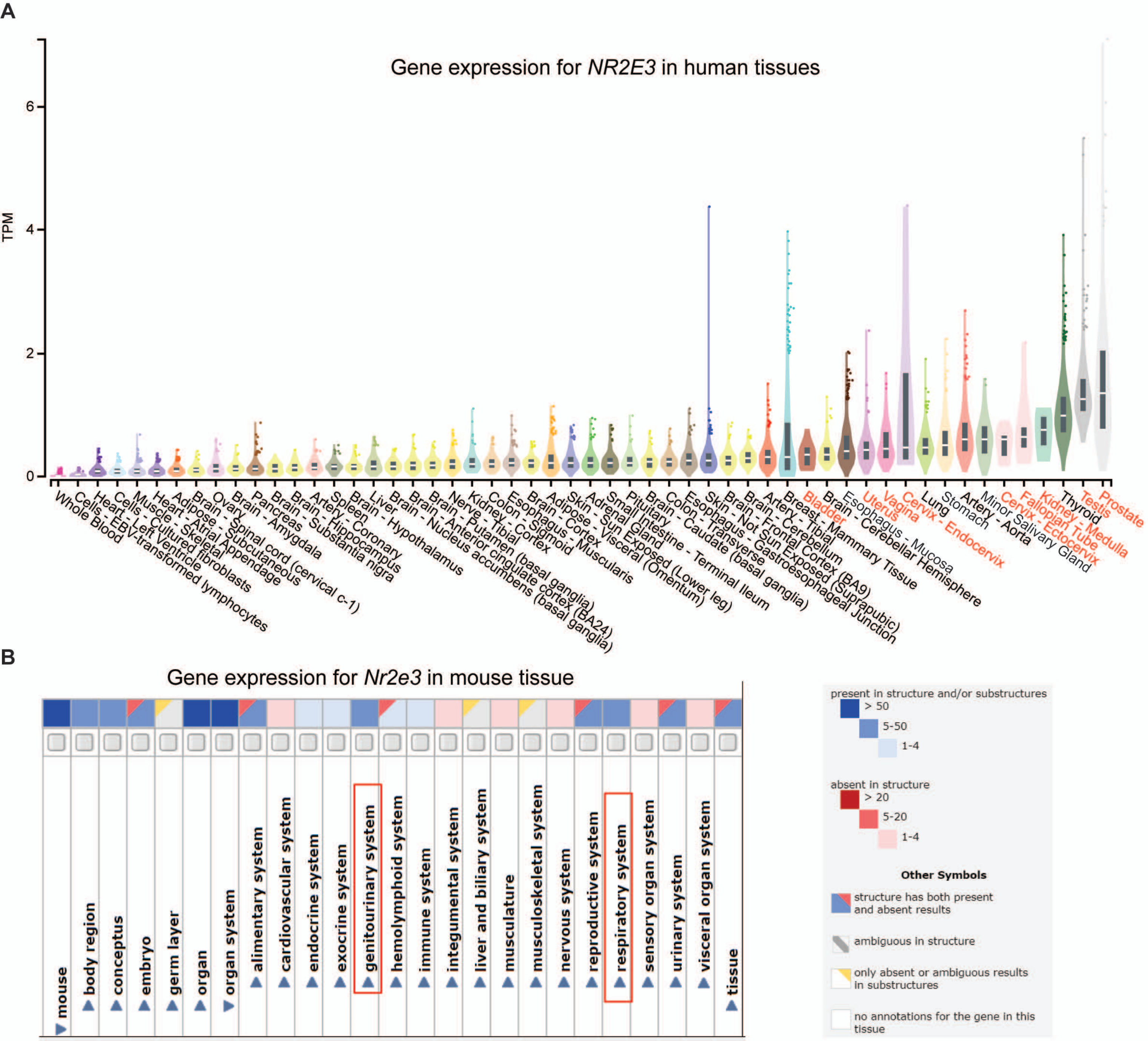
A
Gene expression for NR2E3 in human tissues
TPM
0
2
4
6
Whole Blood
Cells - EBV-transformed lymphocytes
Heart - Left Ventricle
Cells - Cultured fibroblasts
Muscle - Skeletal
Heart - Atrial Appendage
Adipose - Subcutaneous
Brain - Spinal cord (cervical c-1)
Ovary
Brain - Amygdala
Pancreas
Brain - Substantia nigra
Brain - Hippocampus
Artery - Coronary
Spleen
Brain - Hypothalamus
Liver
Brain - Nucleus accumbens (basal ganglia)
Brain - Anterior cingulate cortex (BA24)
Brain - Putamen (basal ganglia)
Nerve - Tibial
Kidney - Cortex
Colon - Sigmoid
Esophagus - Muscularis
Brain - Cortex
Adipose - Visceral (Omentum)
Skin - Sun Exposed (Lower leg)
Adrenal Gland
Small Intestine - Terminal Ileum
Pituitary
Brain - Caudate (basal ganglia)
Colon - Transverse
Esophagus - Gastroesophageal Junction
Skin - Not Sun Exposed (Suprapubic)
Brain - Frontal Cortex (BA9)
Brain - Cerebellum
Artery - Tibial
Breast - Mammary Tissue
Bladder
Brain - Cerebellar Hemisphere
Esophagus - Mucosa
Uterus
Vagina
Cervix - Endocervix
Lung
Stomach
Artery - Aorta
Minor Salivary Gland
Cervix - Ectocervix
Fallopian Tube
Kidney - Medulla
Thyroid
Testis
Prostate
B
Gene expression for Nr2e3 in mouse tissue
mouse
body region
conceptus
embryo
germ layer
organ
organ system
alimentary system
cardiovascular system
endocrine system
exocrine system
genitourinary system
hemolymphoid system
immune system
integumental system
liver and biliary system
musculature
musculoskeletal system
nervous system
reproductive system
respiratory system
sensory organ system
urinary system
visceral organ system
tissue
present in structure and/or substructures
> 50
5-50
1-4
absent in structure
> 20
5-20
1-4
Other Symbols
structure has both present and absent results
ambiguous in structure
only absent or ambiguous results in substructures
no annotations for the gene in this tissue

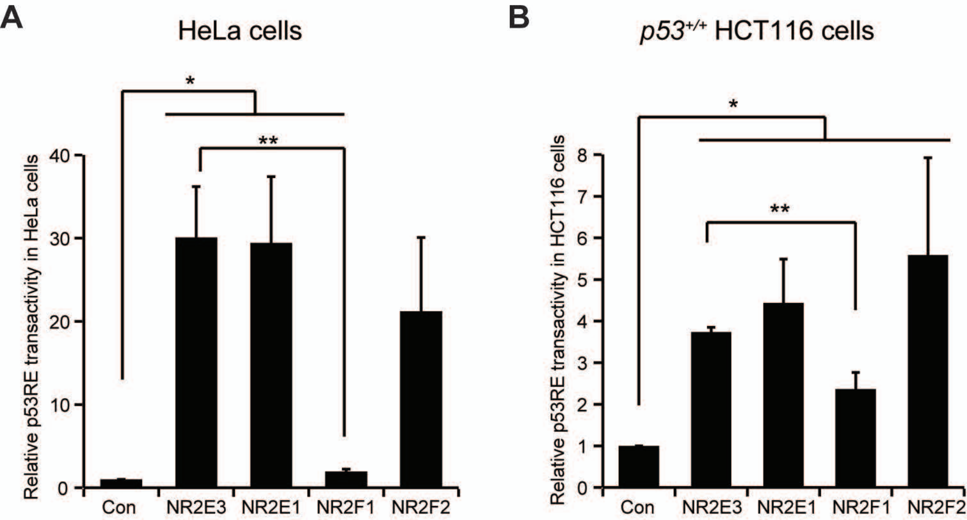
A
HeLa cells
Relative p53RE transactivity in HeLa cells
0
10
20
30
40
*
**
Con
NR2E3
NR2E1
NR2F1
NR2F2
B
p53+/+ HCT116 cells
Relative p53RE transactivity in HCT116 cells
0
1
2
3
4
5
6
7
8
*
**
Con
NR2E3
NR2E1
NR2F1
NR2F2

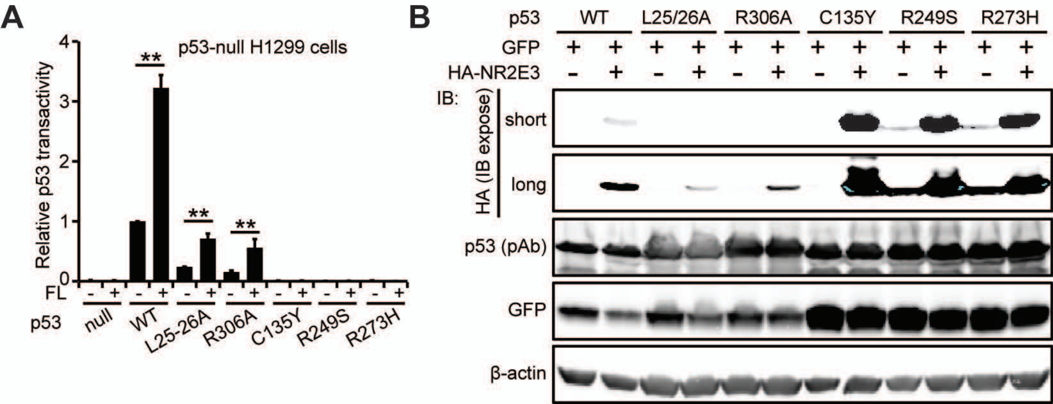
A
p53-null H1299 cells
Relative p53 transactivity
**
FL
p53
null
WT
L25-26A
R306A
C135Y
R249S
R273H
B
p53
WT
L25/26A
R306A
C135Y
R249S
R273H
GFP
HA-NR2E3
IB:
HA (IB expose)
short
long
p53 (pAb)
GFP
β-actin

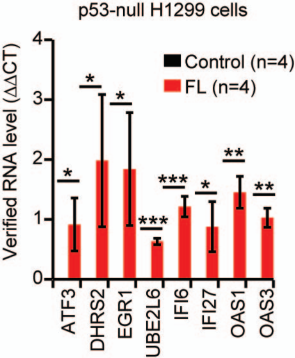
p53-null H1299 cells
Control (n=4)
FL (n=4)
Verified RNA level (ΔΔCT)
0
1
2
3
4
*
*
*
***
***
*
**
**
ATF3
DHRS2
EGR1
UBE2L6
IFI6
IFI27
OAS1
OAS3

A

rd7 vs WT		GSEA name	KO vs WT		GSEA note
NES	FDR q-val		NES	FDR q-val	
1.80	0.009	NRL_DN.V1_UP	1.80	0.031	Positive control: Genes up-regulated in retina cells from $NRL^{-/-}$ and $NRL^{-/-}$; $CRX^{-/-}$ mice.
1.74	0.011	CRX_NRL_DN.V1_UP	1.65	0.028	
1.83	0.019	MODULE_55	1.76	0.020	Stanford Genes in the cancer-promoting module 55
1.53	0.038	BRUINS_UVC_RESPONSE_VIA_TP53_GROUP_A	1.50	0.053	p53-dependent genes in the absence of S389 phosphorylation are similar to $p53^{-/-}$ MEF cells in response to UV-C irradiation

B

BRUINS_UVC_RESPONSE_VIA_TP53_GROUP_A BRUINS_UVC_RESPONSE_VIA_TP53_GROUP_A

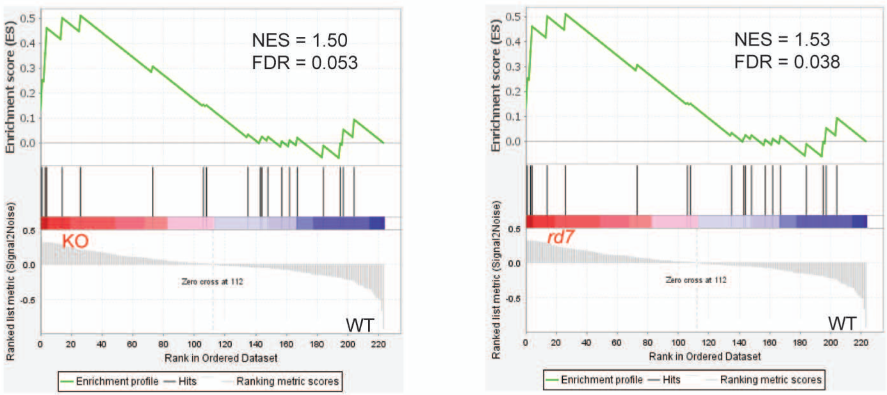

C

MODULE_55 MODULE_55

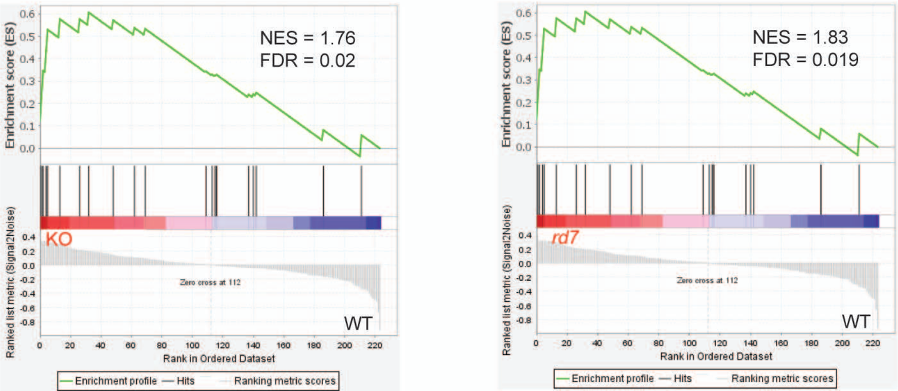

A

DNA Variations		Molecular consequences		Allele origins	
Type	Number	Type	Number	Type	Number
Deletion	47	Frameshift	30	Germline	335
Duplication	23	Missense	226	De novo	3
Indel	1	Nonsense	16	Somatic	0
Insertion	16	Splice site	13		
Single nucleotide	525	UTR	27		

B

Report resources		Review status		Clinical significances	
Type	Number	Type	Number	Type	Number
Research	19	Practice guideline	0	Conflicting interpretation	33
Literature only	10	Expert panel	0	Benign	22
Clinical testing	338	Multiple submitters	77	Likely benign	206
		Single submitter	469	Uncertain significance	253
		At least one star	579	Likely pathogenic	25
				Pathogenic	82

C

Mutant	SNP	Position	DNA change	Allele frequency	Effects	Reported	Cancer involved	Frequency in cancer
R76W	rs104894492	15: 71811590	C>T	0.00002629	Pathogenic	Yes		
G88V	rs1278137915	15: 71811783	G>T	0.00000000	Pathogenic	Yes		
R97H	rs1489149705	15: 71811810	G>A	0.00001971	Pathogenic	Yes	UCEC	2 / 512 (0.39%)
E121K	rs146403122	15: 71811966	G>A	0.00231300	Benign	Yes		
V302I	rs1805025	15: 71813545	G>A	0.00150400	Benign	Yes		
M407K	rs1303613101	15: 71817671	T>A	0.00000806	n/a	Yes		

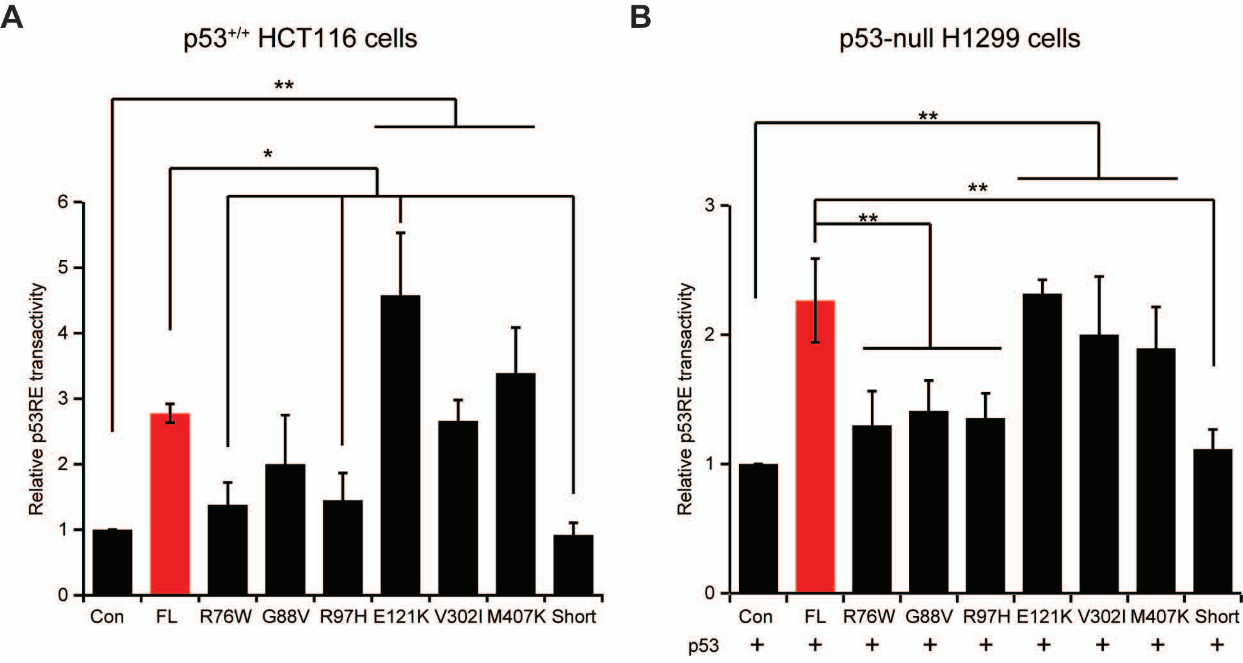
A
p53+/+ HCT116 cells
Relative p53RE transactivity
0
1
2
3
4
5
6
**
*
Con
FL
R76W
G88V
R97H
E121K
V302I
M407K
Short
B
p53-null H1299 cells
Relative p53RE transactivity
0
1
2
3
**
**
**
Con
FL
R76W
G88V
R97H
E121K
V302I
M407K
Short
p53
+
+
+
+
+
+
+
+
+

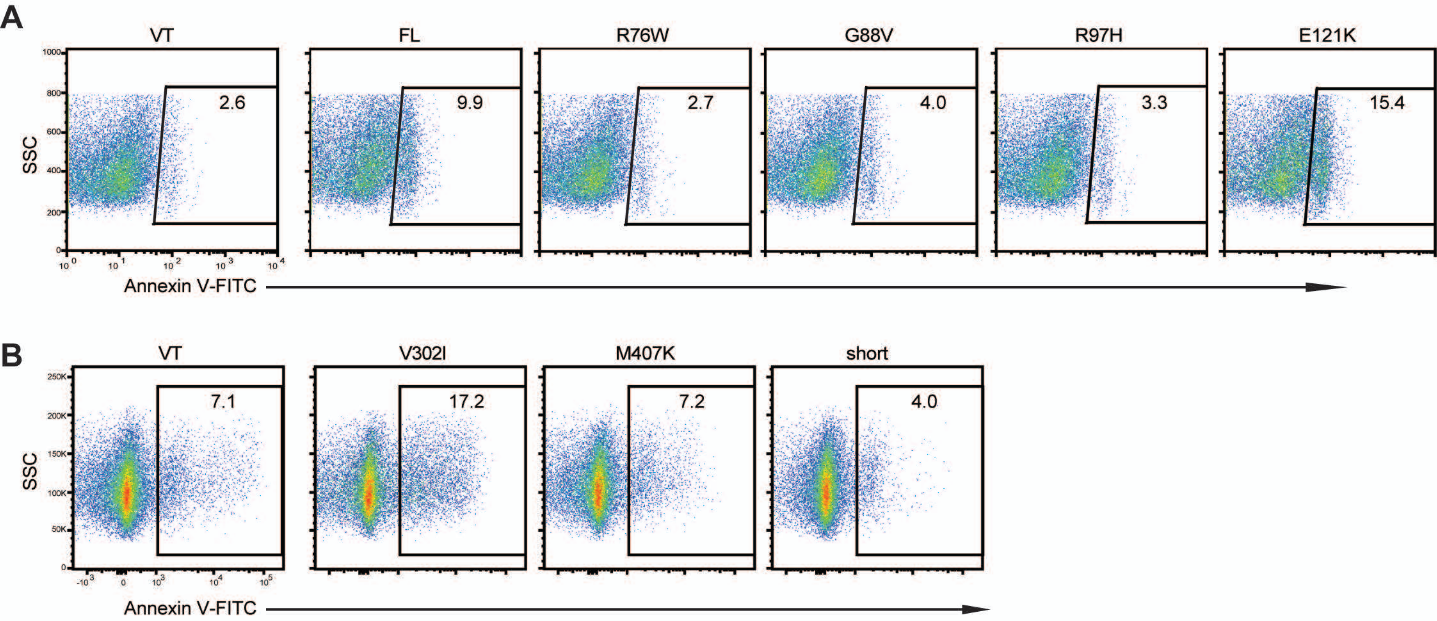
A
VT
FL
R76W
G88V
R97H
E121K
2.6
9.9
2.7
4.0
3.3
15.4
SSC
Annexin V-FITC
B
VT
V302I
M407K
short
7.1
17.2
7.2
4.0
SSC
Annexin V-FITC

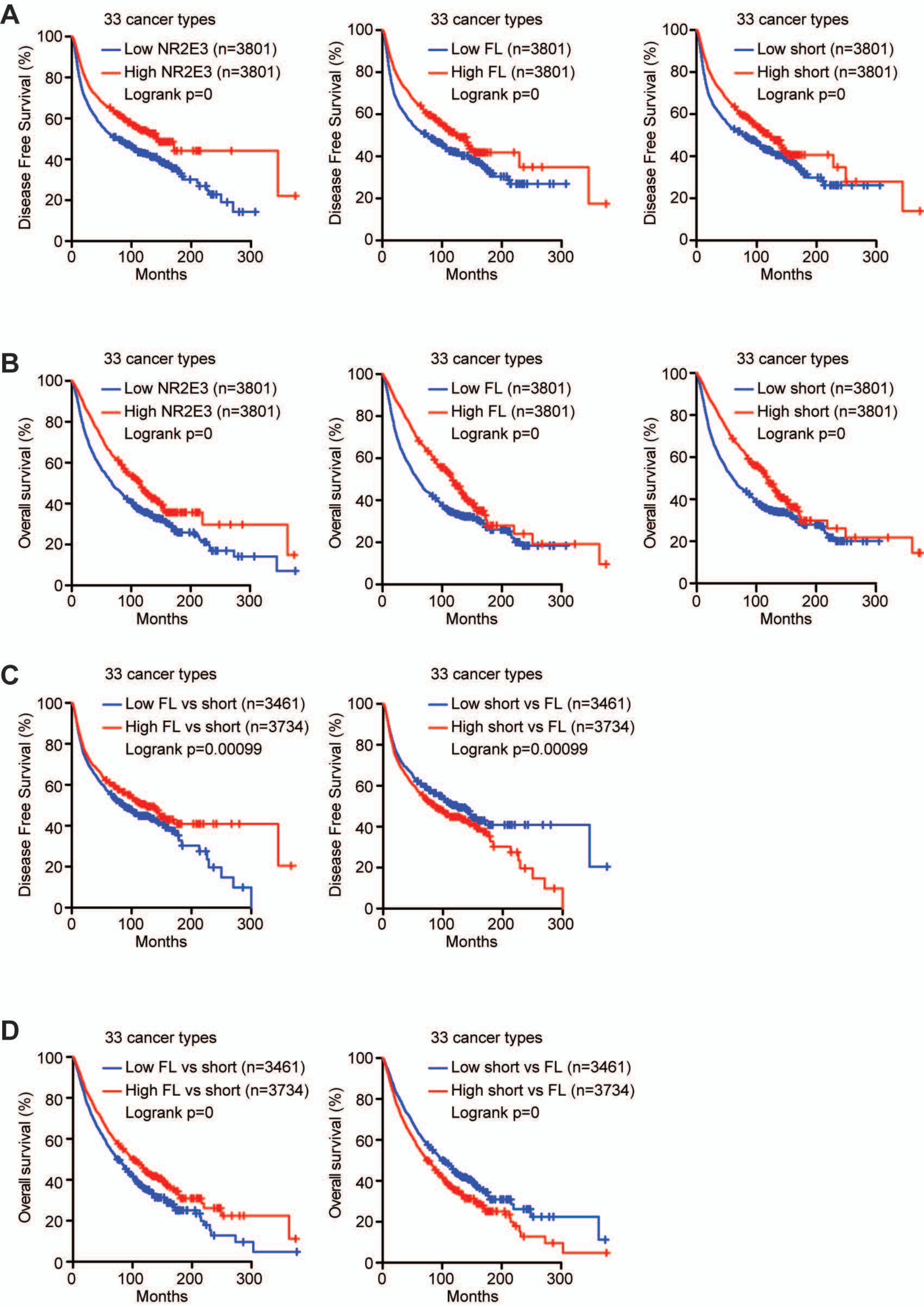
A
B
C
D
33 cancer types
Low NR2E3 (n=3801)
High NR2E3 (n=3801)
Low FL (n=3801)
High FL (n=3801)
Low short (n=3801)
High short (n=3801)
Logrank p=0
Low FL vs short (n=3461)
High FL vs short (n=3734)
Low short vs FL (n=3461)
High short vs FL (n=3734)
Logrank p=0.00099
Disease Free Survival (%)
Overall survival (%)
Months

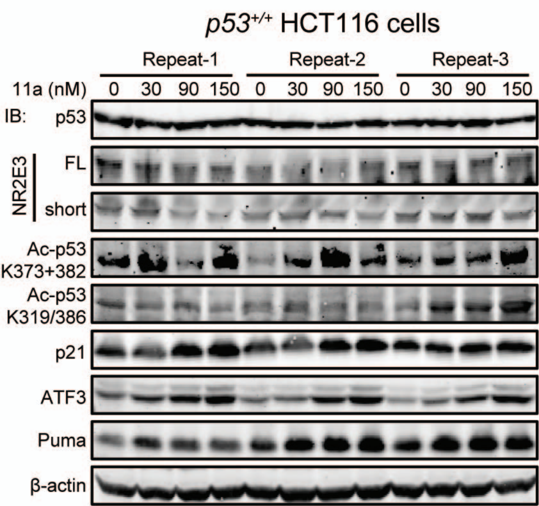
p53+/+ HCT116 cells
Repeat-1
Repeat-2
Repeat-3
11a (nM)
0 30 90 150 0 30 90 150 0 30 90 150
IB:
p53
NR2E3
FL
short
Ac-p53 K373+382
Ac-p53 K319/386
p21
ATF3
Puma
β-actin

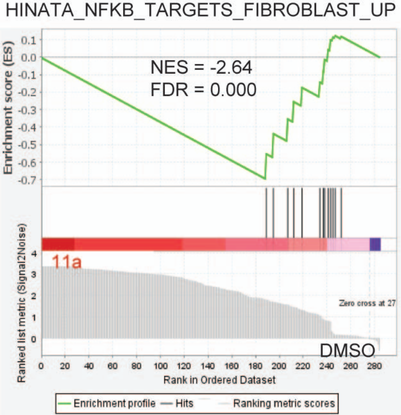
HINATA_NFKB_TARGETS_FIBROBLAST_UP
Enrichment score (ES)
NES = -2.64
FDR = 0.000
11a
Ranked list metric (Signal2Noise)
Zero cross at 27
DMSO
Rank in Ordered Dataset
Enrichment profile
Hits
Ranking metric scores

A

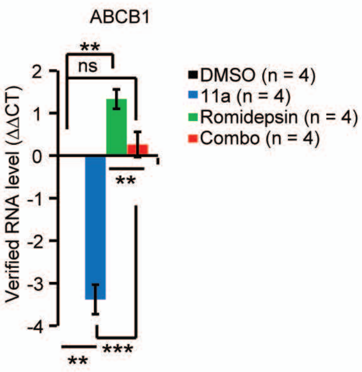

B

GSEA (Hallmark)	11a vs DMSO	Romidepsin vs DMSO	Combo vs DMSO	Combo vs Romidepsin	Combo vs 11a	Interaction mode
DNA_REPAIR	NES = -1.285	NES = -2.029	NES = -1.986	NES = -1.142	NES = -1.438	sum-up
	FDR = 0.091	FDR = 0.002	FDR = 0.000	FDR = 0.211	FDR = 0.026	
G2M_CHECKPOINT	NES = -1.133	NES = -4.100	NES = -2.953	NES = -1.251	NES = -2.609	sum-up
	FDR = 0.249	FDR = 0.000	FDR = 0.000	FDR = 0.108	FDR = 0.000	
E2F_TARGETS	NES = -1.476	Not enriched	NES = -3.065	NES = -1.756	NES = -2.778	helper
	FDR = 0.025		FDR = 0.000	FDR = 0.003	FDR = 0.000	
ROS_PATHWAY	NES = -1.329	NES = -1.141	NES = -1.681	NES = -1.841	NES = -1.586	sum-up
	FDR = 0.065	FDR = 0.160	FDR = 0.004	FDR = 0.002	FDR = 0.007	
ANGIOGENESIS	NES = -1.373	NES = 1.181	NES = -1.140	NES = -1.567	Not enriched	antagonism
	FDR = 0.052	FDR = 0.195	FDR = 0.215	FDR = 0.011		
MYC_TARGETS_V2	NES = 1.253	NES = -3.383	NES = -2.972	NES = -1.279	NES = -3.160	antagonism
	FDR = 0.114	FDR = 0.000	FDR = 0.000	FDR = 0.088	FDR = 0.000	
GLYCOLYSIS	NES = -1.608	NES = 1.191	Not enriched	NES = -1.364	NES = 1.192	antagonism
	FDR = 0.011	FDR = 0.185		FDR = 0.052	FDR = 0.229	
