# Supplementary material for: Orphan nuclear receptor NR2E3 is a new molecular vulnerability in solid tumors by activating p53
Source: Cell Death Dis. 2025 Jan 14;16(1):15. doi: 10.1038/s41419-025-07337-1 (PMC11733144; doi:10.1038/s41419-025-07337-1)
Supplement: Supplementary file 3 — Full and uncropped Western blot images [file 41419_2025_7337_MOESM3_ESM.pdf]

Figure 1A

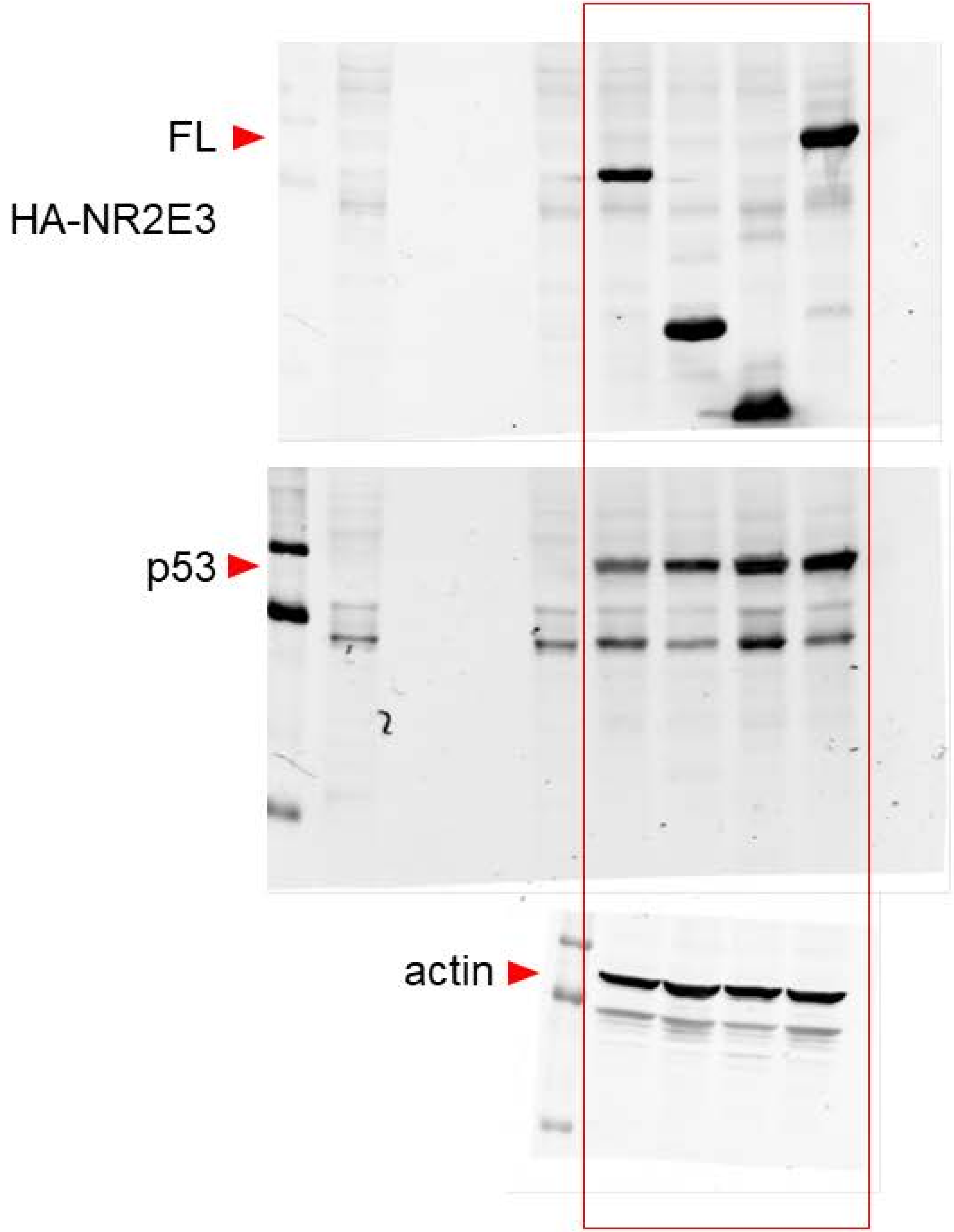

Figure 1G

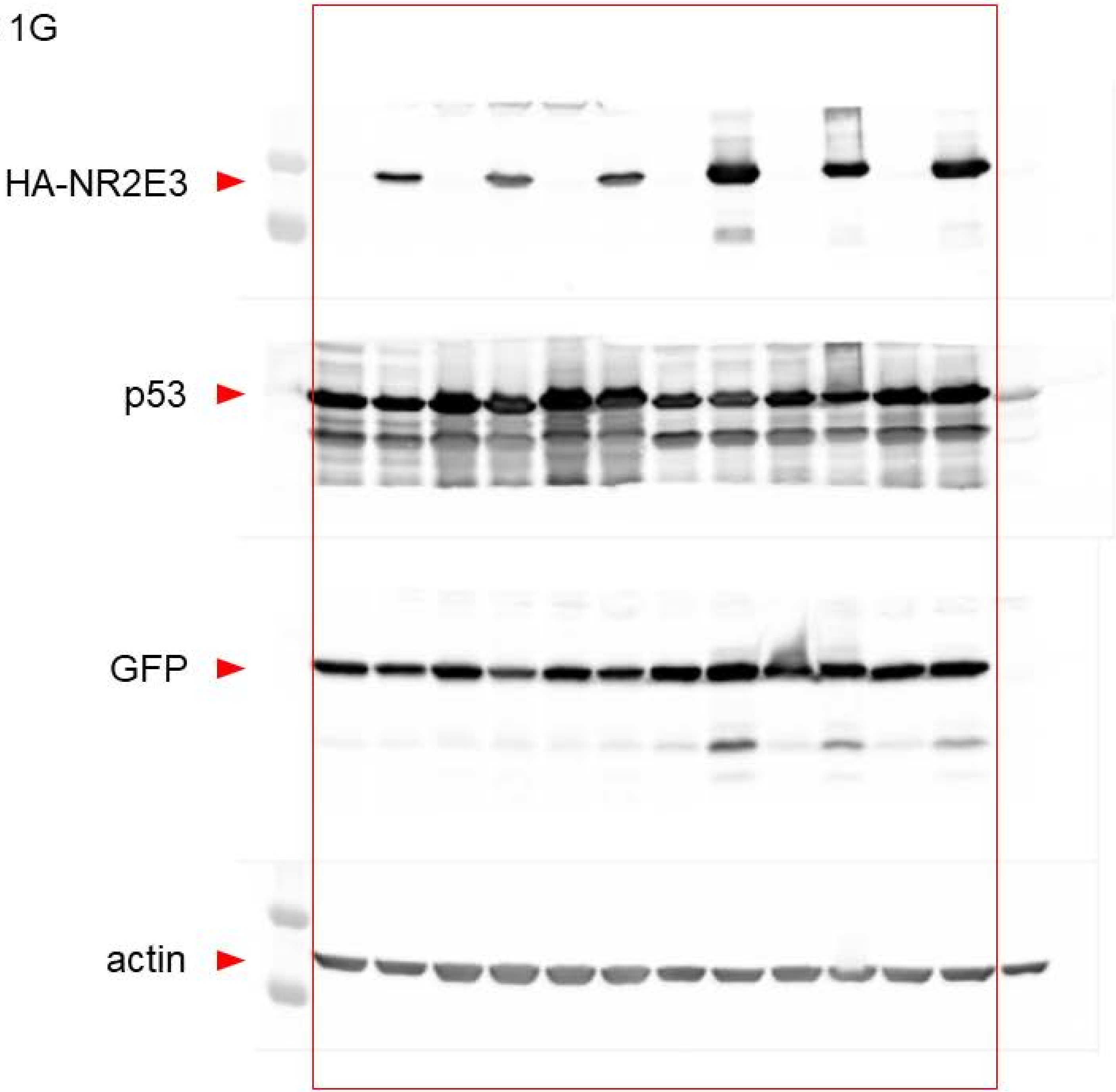

Figure 2B

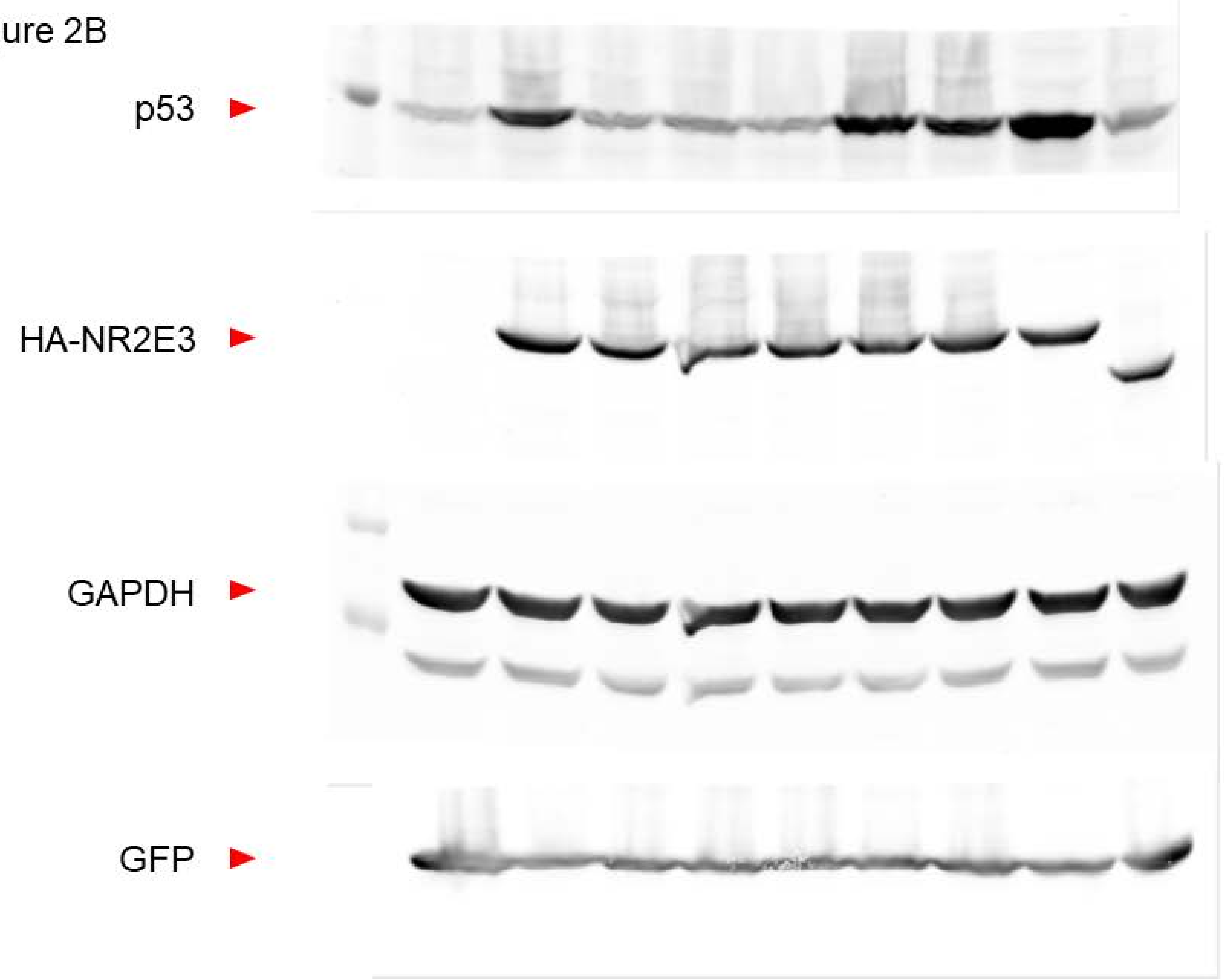

Figure 3B

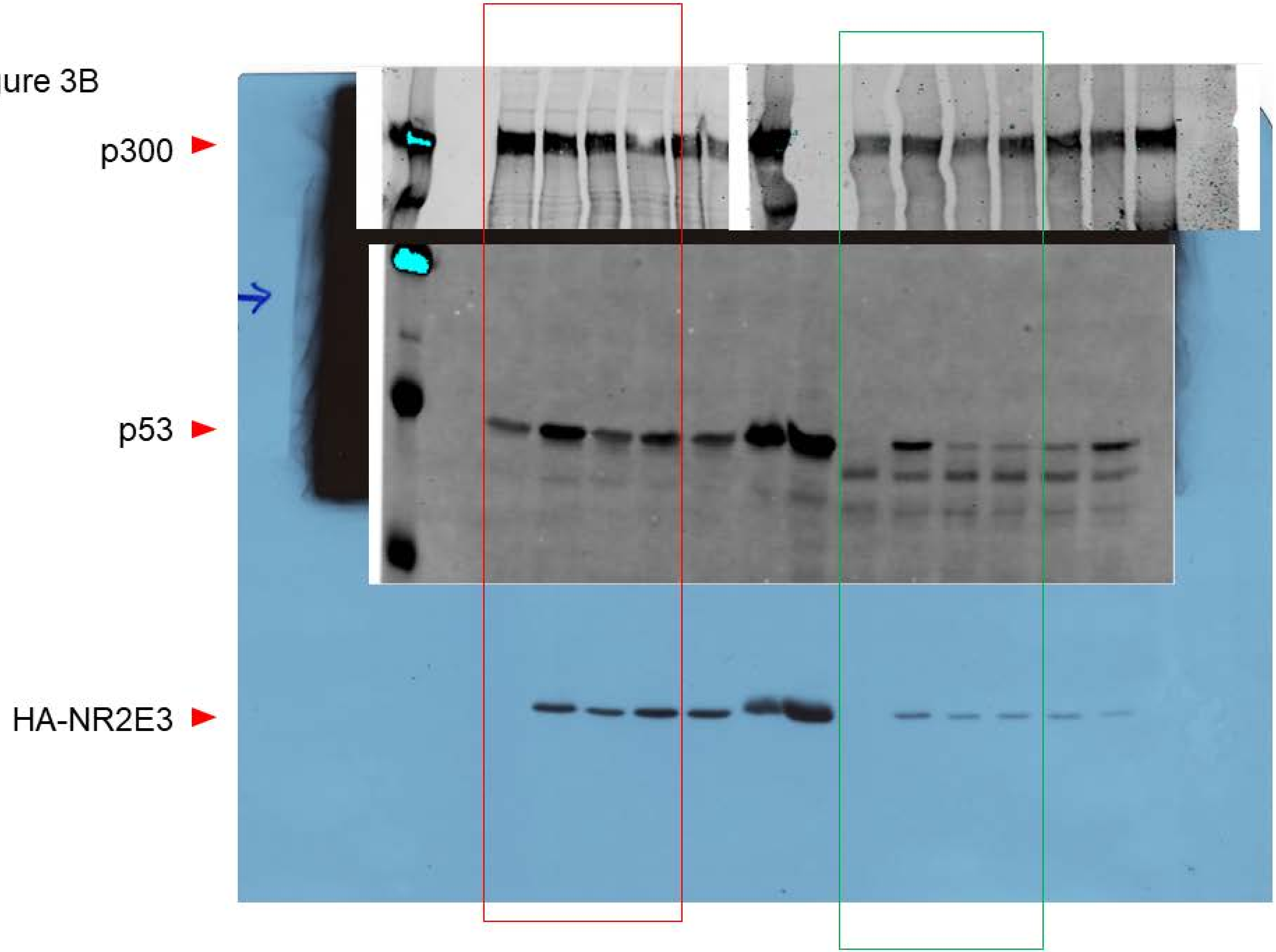

Figure 3C

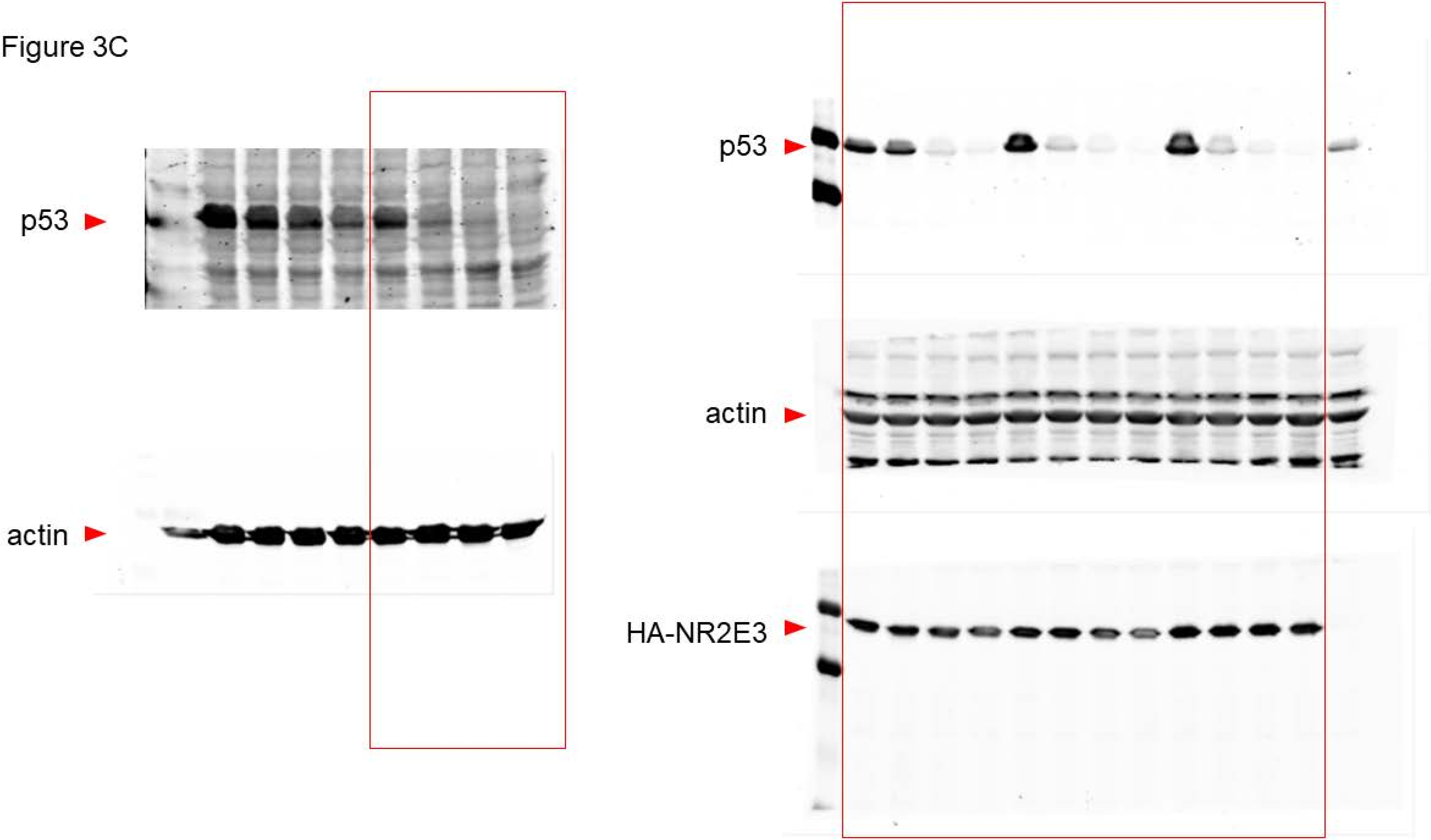

Figure 3J

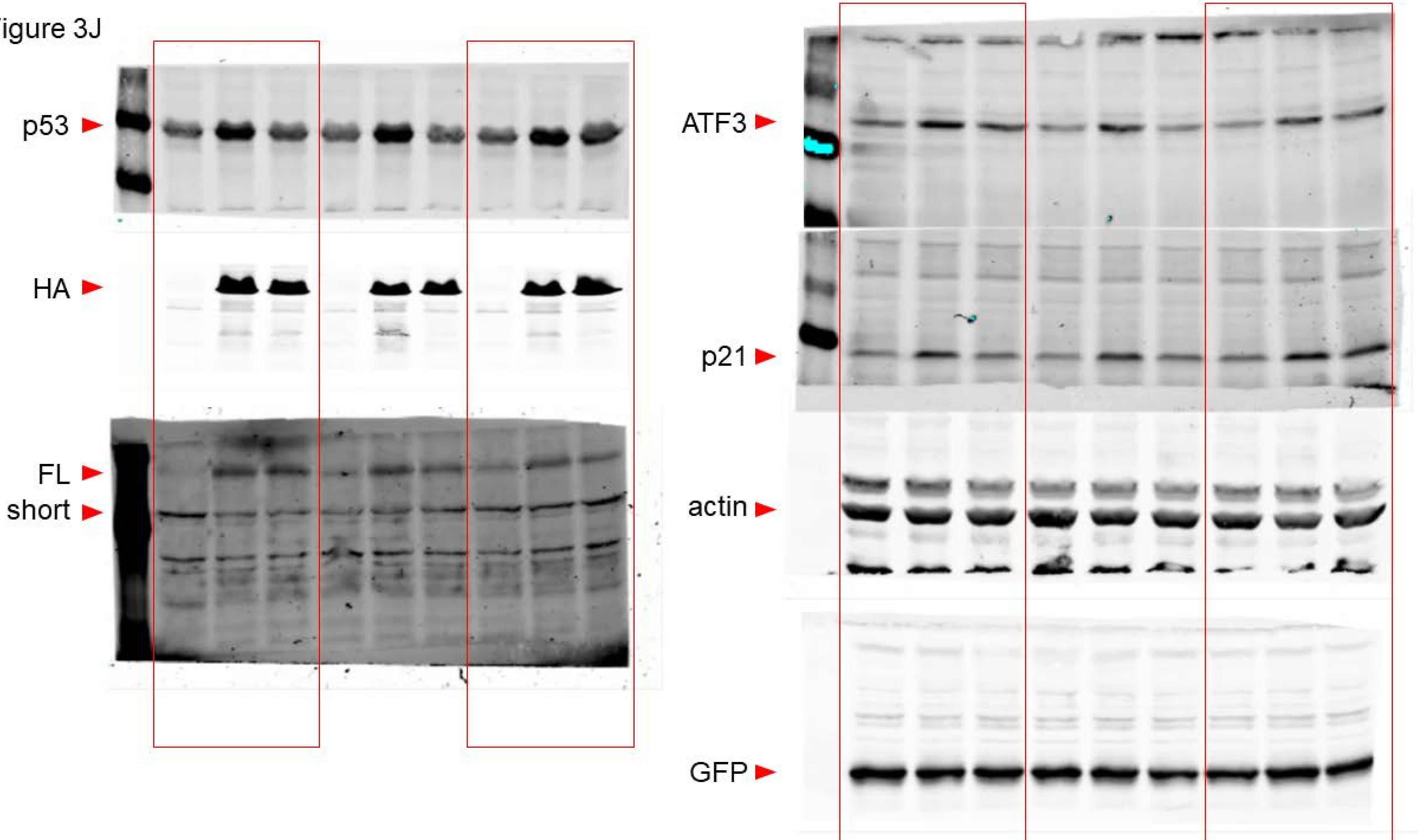

Figure 3M

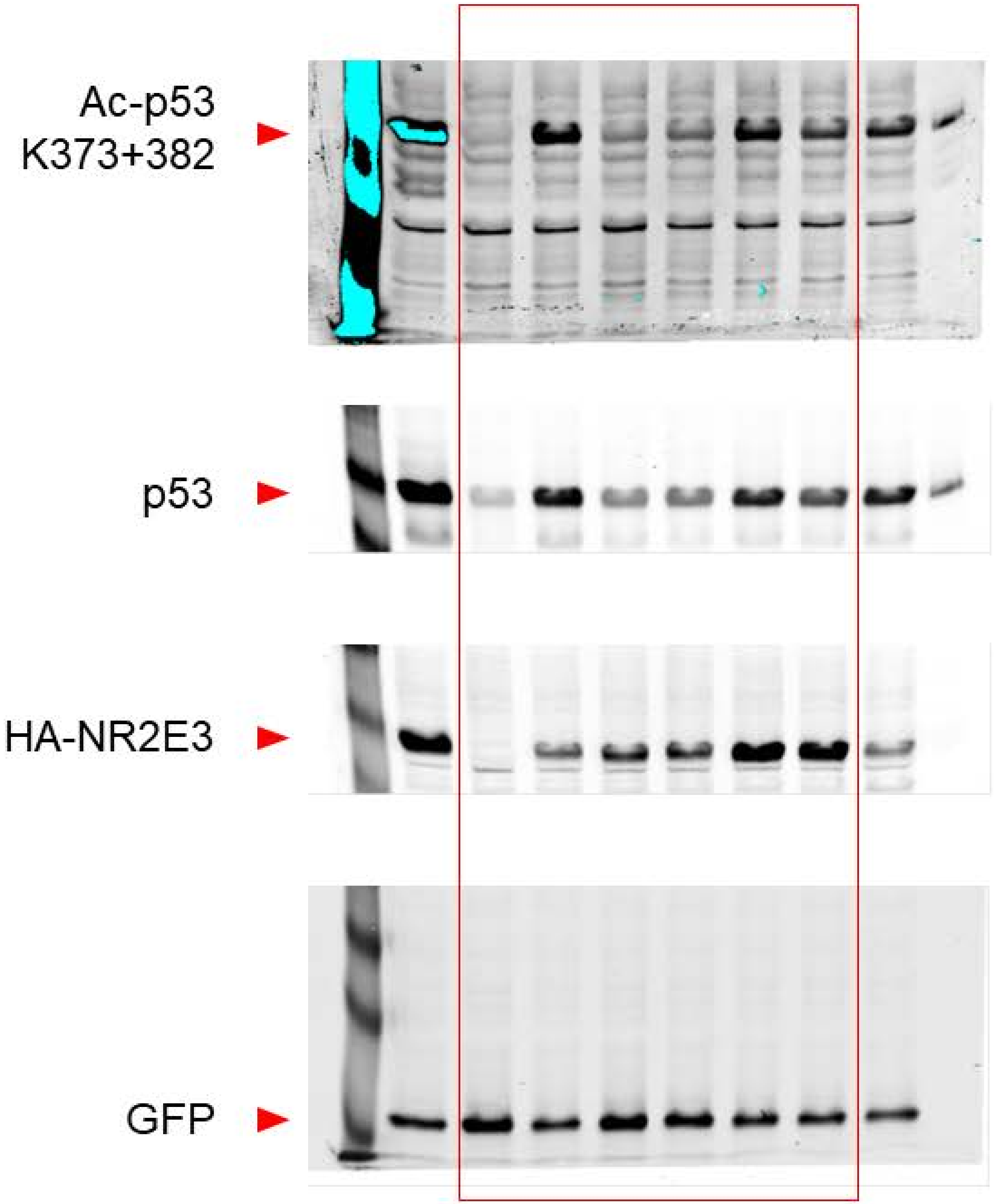

Figure 4D

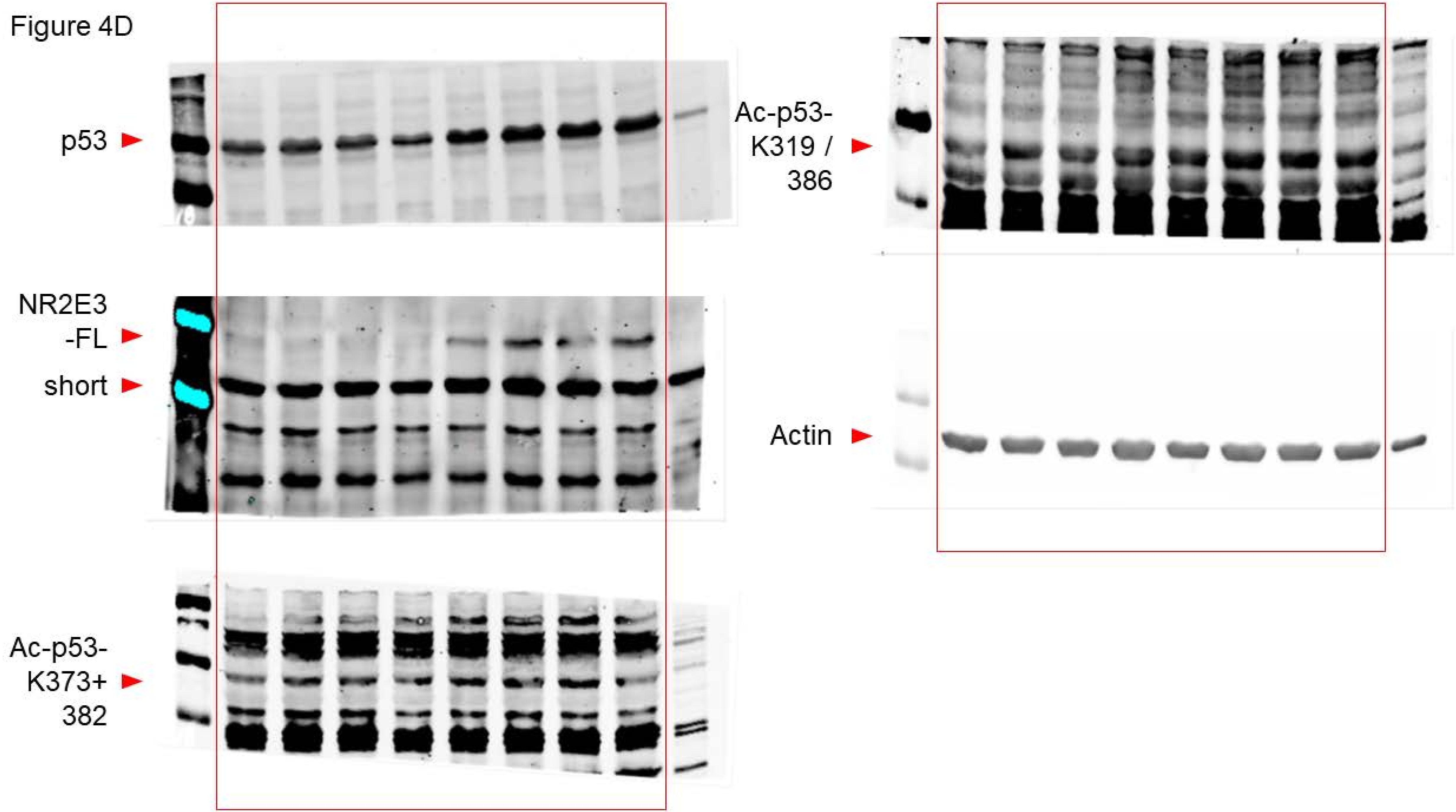

Figure 4E

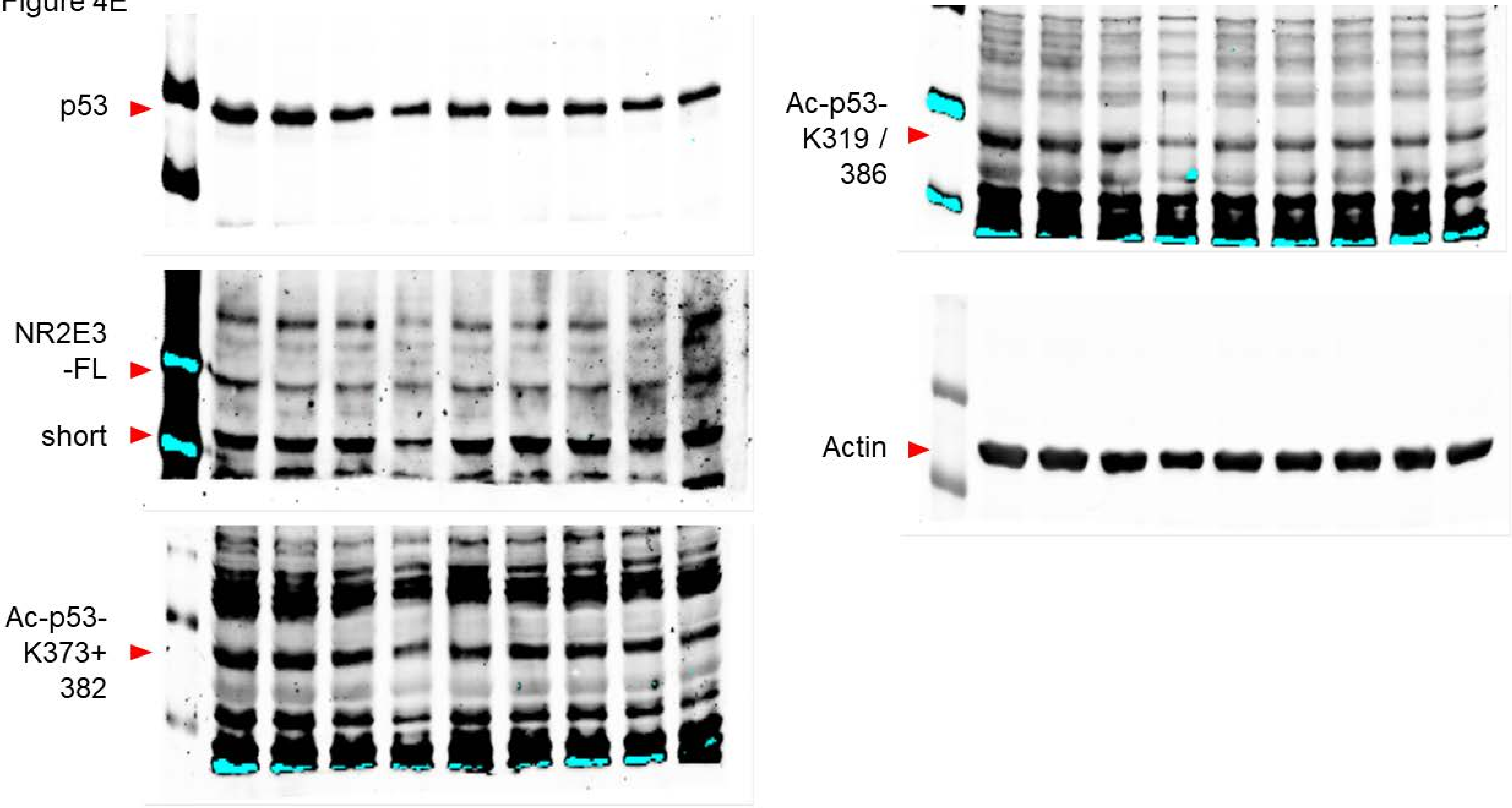

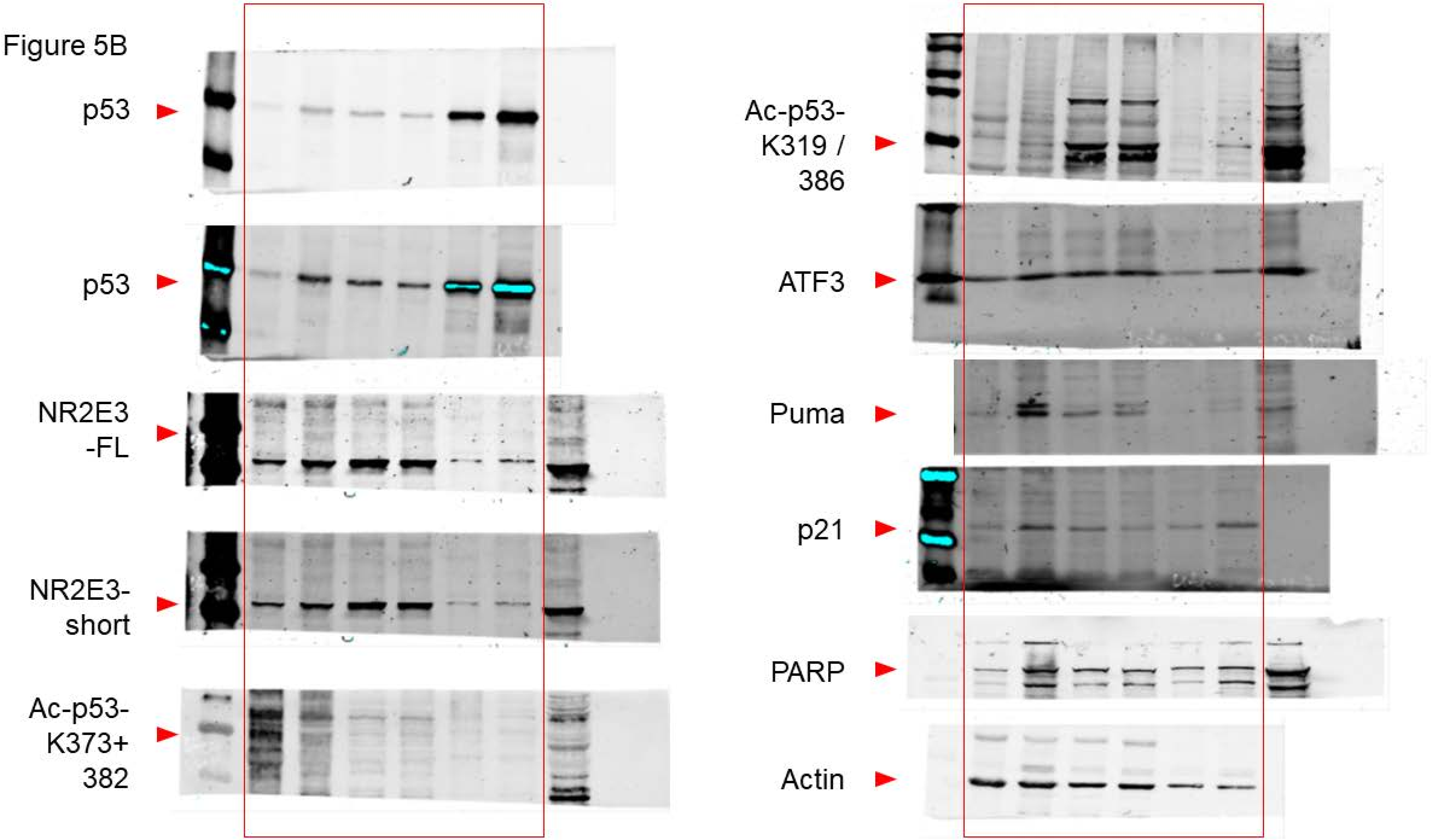
Figure 5B
p53
p53
NR2E3
-FL
NR2E3-
short
Ac-p53-
K373+
382
Ac-p53-
K319 /
386
ATF3
Puma
p21
PARP
Actin

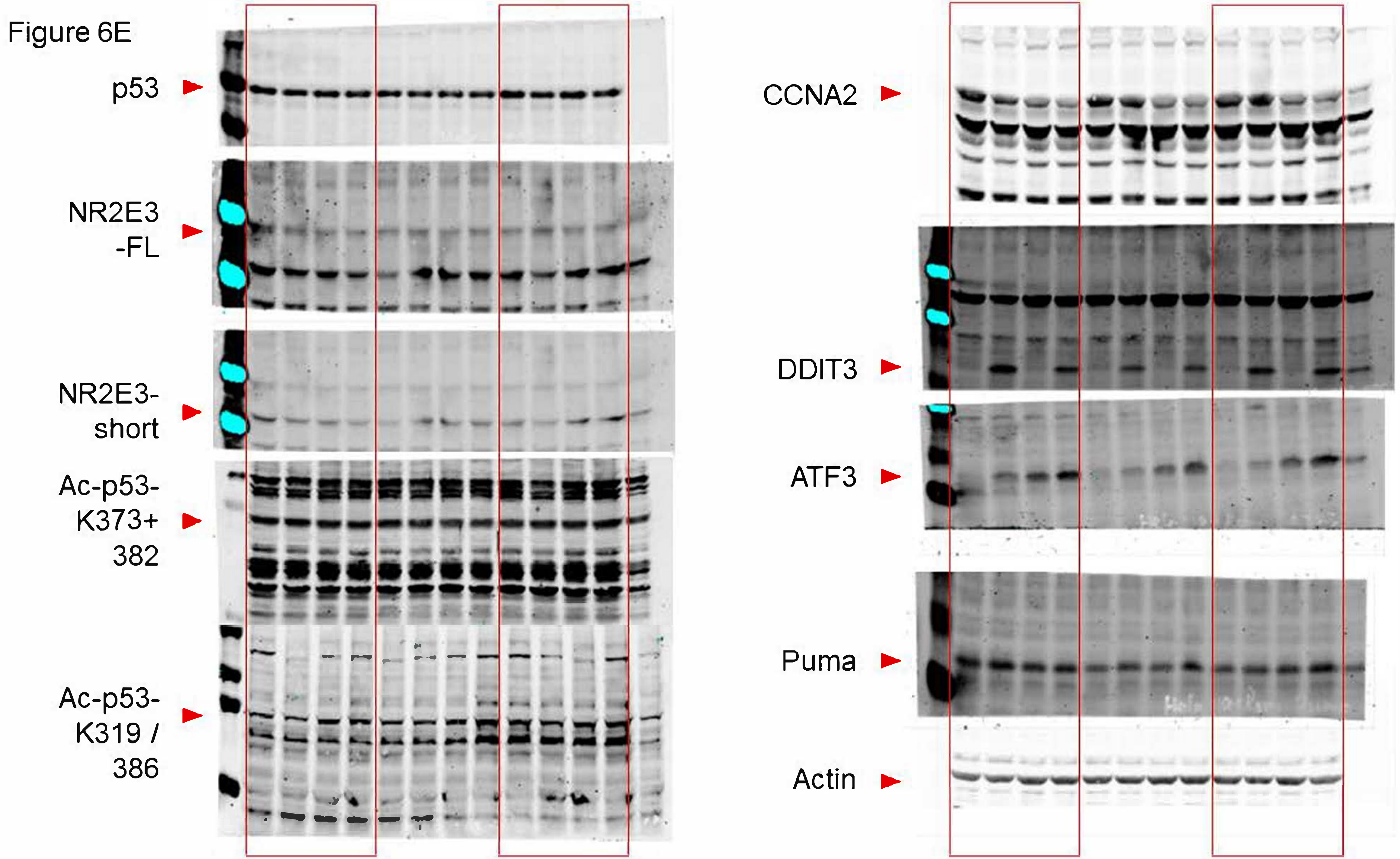
Figure 6E
p53
NR2E3
-FL
NR2E3-
short
Ac-p53-
K373+
382
Ac-p53-
K319 /
386
CCNA2
DDIT3
ATF3
Puma
Actin

Figure S3B

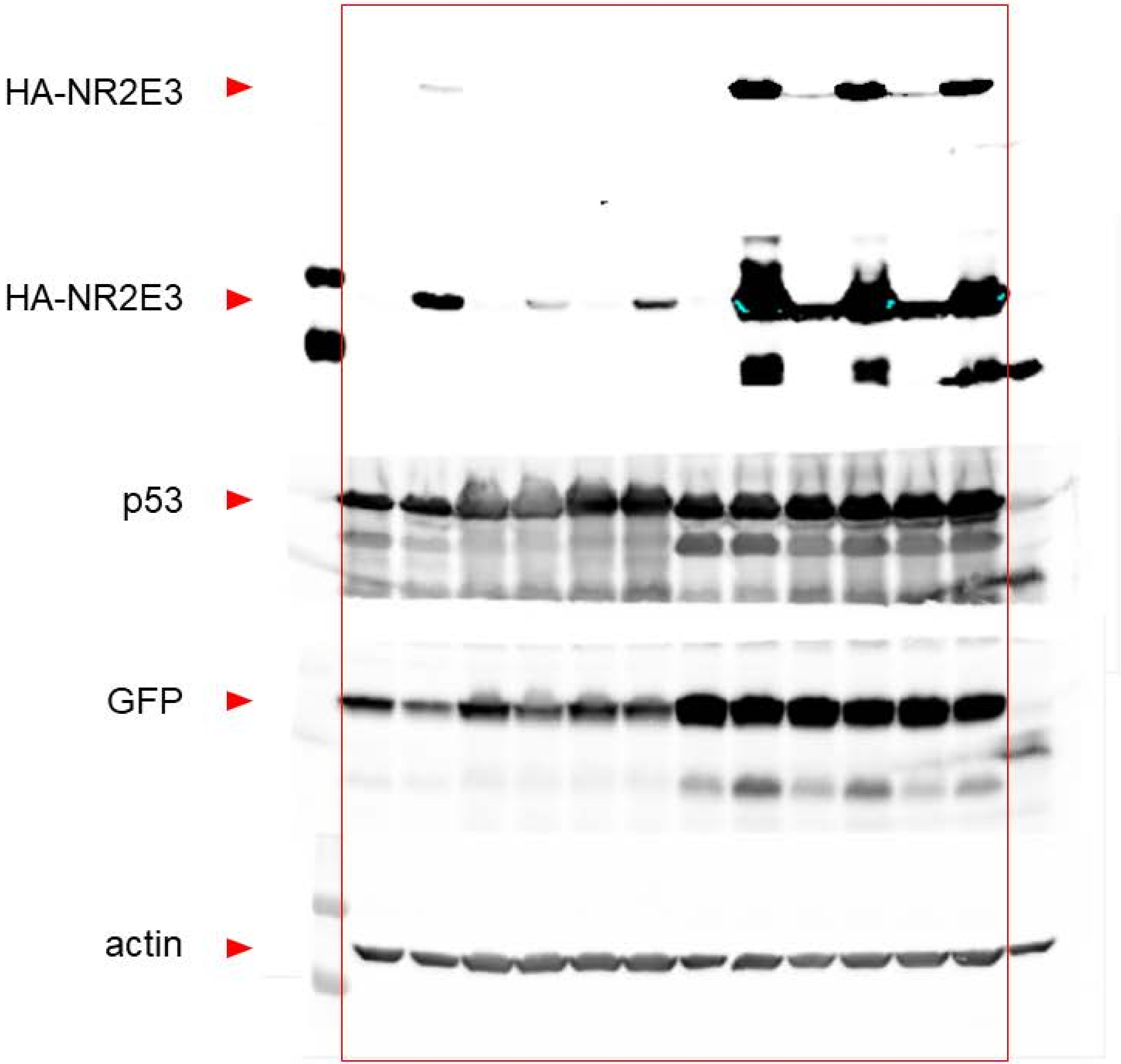

Figure S10

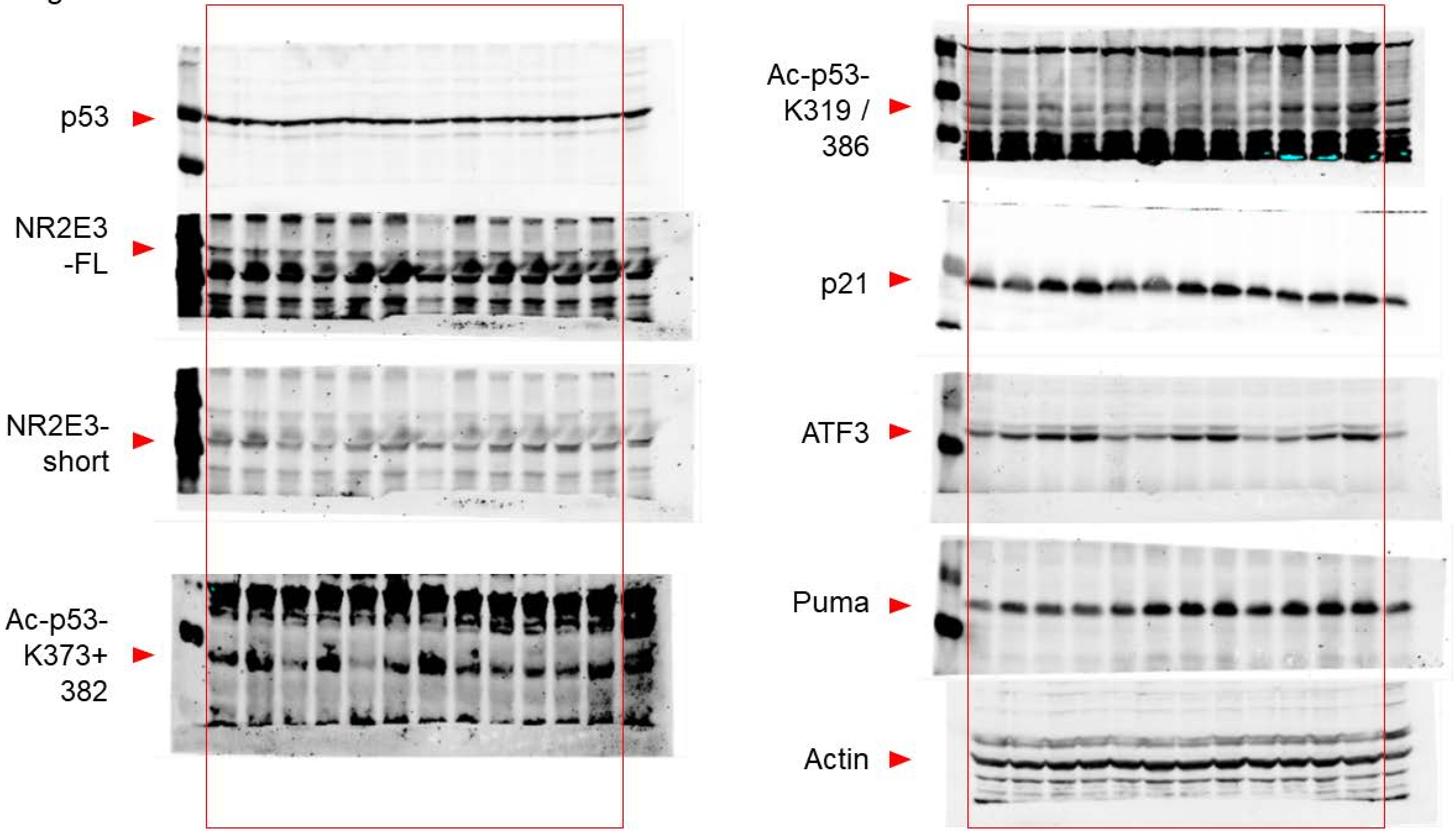
p53
NR2E3
-FL
NR2E3-
short
Ac-p53-
K373+
382
Ac-p53-
K319 /
386
p21
ATF3
Puma
Actin
